# Supplementary material for: Sustainable Synthesis, Antiproliferative and Acetylcholinesterase Inhibition of 1,4- and 1,2-Naphthoquinone Derivatives
Source: Molecules. 2023 Jan 27;28(3):1232. doi: 10.3390/molecules28031232 (PMC9919139; doi:10.3390/molecules28031232)
Supplement: Supplementary file 1 [file molecules-28-01232-s001.zip › molecules-2170620-supplementary.pdf]

molecules

MDPI

Supplementary Materials

Sustainable synthesis, antiproliferative and acetylcholinesterase inhibition of 1,4- and 1,2-naphthoquinone derivatives

Rafaela G. Cabral [1,2], Gonçalo Viegas [1], Rita Pacheco [1,2], Ana Catarina Sousa [1,2]*and Maria Paula Robalo [1,2]*

[1] Departamento de Engenharia Química, Instituto Superior de Engenharia de Lisboa, Instituto Politécnico de Lisboa,1959-007 Lisboa, Portugal;

[2] Centro de Química Estrutural, Institute of Molecular Sciences, Instituto Superior Técnico, Universidade de Lisboa 1049-001 Lisboa, Portugal.;

* Correspondence: MPR: mprobalo@deq.isel.ipl.pt and ACS: acsousa@deq.isel.ipl.pt.

Contents

1. HPLC profiles S2

2. Electrochemical studies S3

3. *In silico* analysis S9

4. *In vitro* cytotoxicity against human hepatocarcinoma cell line HepG2 S10

5. Inhibition of acetylcholinesterase activity S12

6. Molecular docking studies S13

7. 1D and 2D NMR spectra S14

8. ESI/MS spectra S31

9. HRESI/TOFMS spectra S40

1. HPLC profiles

A)

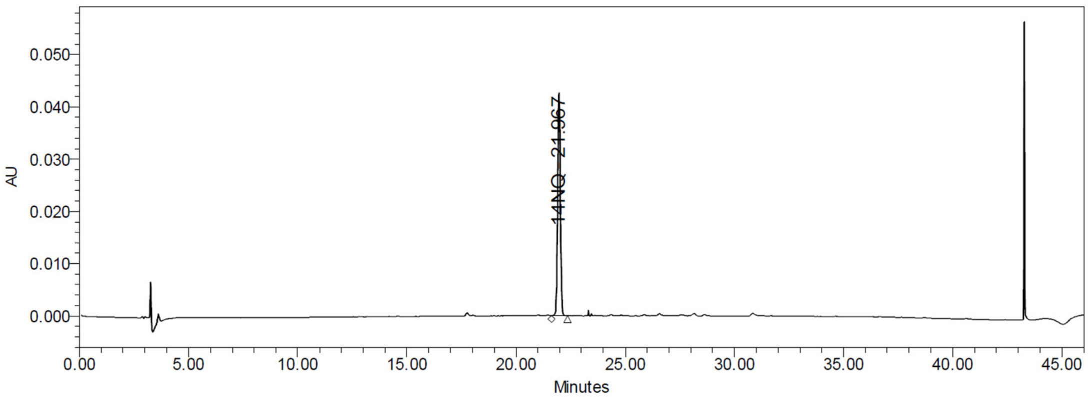

B)

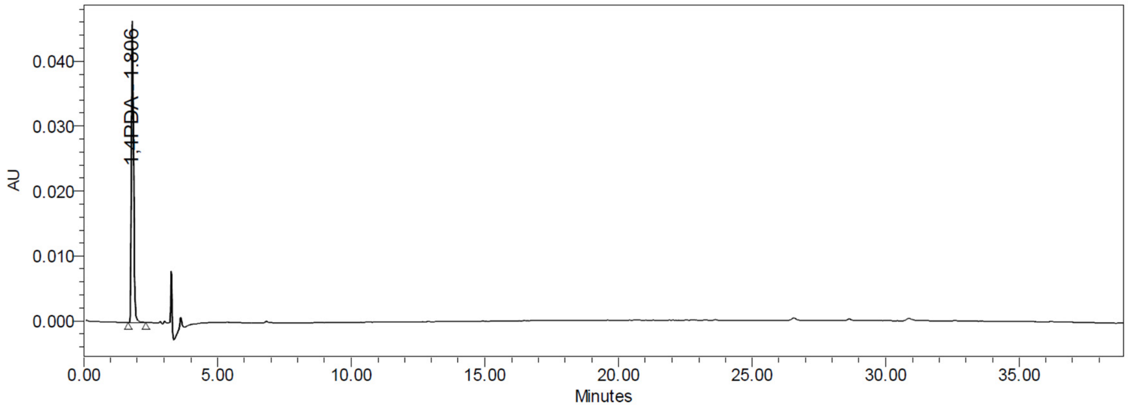

C)

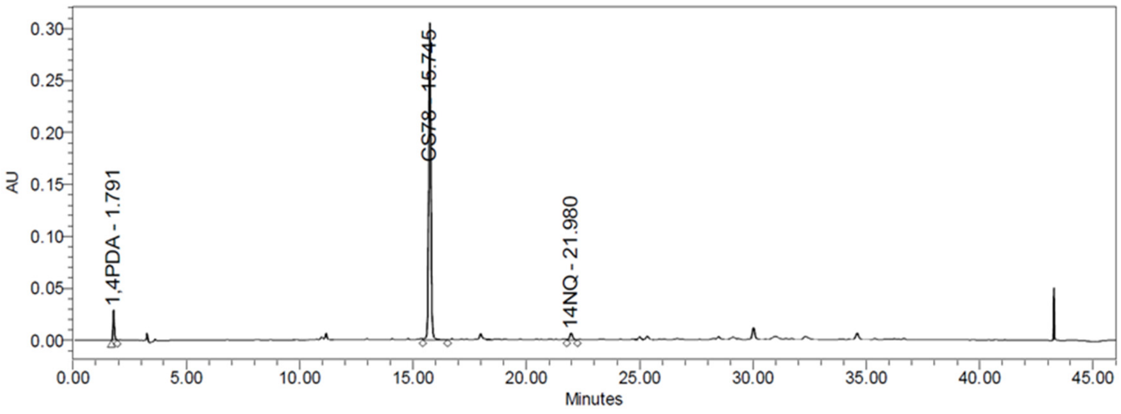

Figure S1. HPLC profiles of ***A)*** 1,4-NQ; ***B)*** 1,4-PDA and ***C)*** MA synthesis of ***3a***, at optimized conditions (entry 15. Table 1).

2. Electrochemical studies

Table S1. Electrochemical parameters for 1,4-NQ and derivatives in acetonitrile (potentials are given *vs* SCE; scan rate: 100 mV.s^{-1}).

Compound	E_{pa} (V)	E_{pc} (V)	$E_{1/2}$ (V)	E_{pa}. E_{pc} (mV)	I_{pa}/I_{pc}
1	-0.62	-0.70	-0.66	80	0.9
	-1.02	-1.36	--	--	--
3a	0.72	0.44	--	--	--
	-0.78	-0.86	-0.82	80	0.9
	-1.27	-1.42	--	--	--
3b	1,04	--	--	--	--
	-0,77	-0.84	-0.81	70	1.0
3c	1,21	--	--	--	--
	- 0,79	-0.86	-0.82	70	1.0
	-1,22	-1.34	-1.28	120	1.0
3d	-0.66	-0.74	-0.70	80	1.0
3e	1,55	--	--	--	--
	0.74	--	--	--	--
	-0.74	-0.98	-0.95	70	1.0
	-1.37	-1.47	-1.42	100	1.0
3f	1,40	--	--	--	--
	-0,90	-0.97	-0.94	70	1.0

Table S2. Electrochemical parameters for 1,2-NQ and derivatives in acetonitrile (potentials are given *vs* SCE; scan rate: 0.100 V.s^{-1}).

Compound	E_{pa} (V)	E_{pc} (V)	$E_{1/2}$ (V)	E_{pa}. E_{pc} (mV)	I_{pa}/I_{pc}
2	-0.44	-0.55	-0.50	110	1.0
	-0.84	-0.98	-0.91	140	1.0
4a	0.81	--	--	--	--
	-0.47	-0.67	--	--	--
4b	1,16	--	--	--	--
	0,93	--	--	--	--
	-0,36	-0.61	--	--	--
4c	1.07	--	--	--	--
	-0.44	-0.67	--	--	--
	-1.30	-1.58	--	--	--
4d	0.76	--	--	--	--
	-0.33	-0.47	--	--	--
	--	-0.84	--	--	--
4g	0.92	--	--	--	--
	0.75	--	--	--	--
	-0.35	-0.63	--	--	--
	--	-1.43	--	--	--
4h	1.39	--	--	--	--
	1.08	--	--	--	--
	--	0.13	--	--	--
	-0.15	-0.57	--	--	--
	--	-0.81	--	--	--
	-0.75	-0.98	--	--	--
4e	1.48	--	--	--	--
	1.01	--	--	--	--
	-0.63	-0.78	-0.71	150	--
4f	1.38	--	--	--	--
	0.91	--	--	--	--
	--	-0.52	--	--	--
	-0.57	-0.69	-0.63	120	0.9

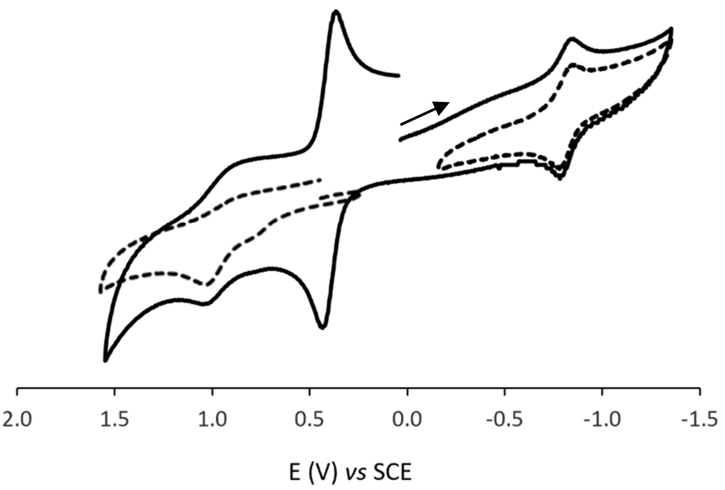

Figure S2. Cyclic voltammogram of ***3b*** in ACN + $TBAPF_6$ showing the ferrocene redox pair (black line) and the isolated redox processes (dashed line) at scan rate of 100 mV.s^{-1}.

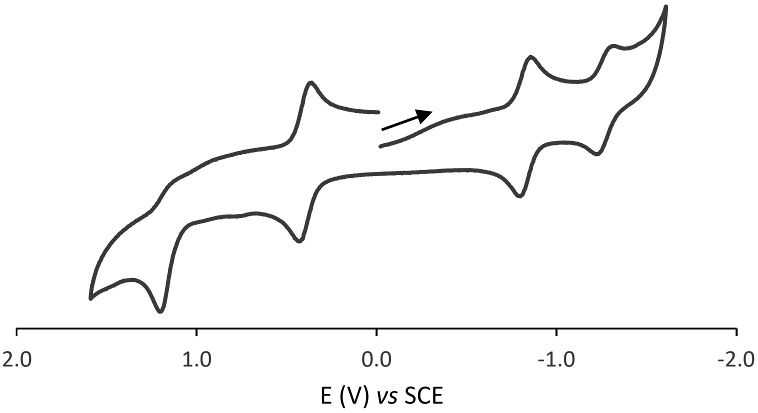

Figure S3. Cyclic voltammogram of ***3c*** in ACN + $TBAPF_6$, showing the ferrocene redox pair at scan rate of 100 mV.s^{-1}.

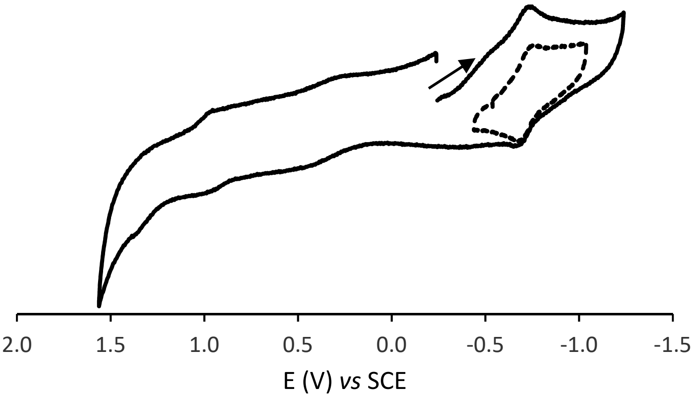

Figure S4. Cyclic voltammogram of ***3d*** in ACN + $TBAPF_6$ (black line) showing the isolated redox process (dashed line) at scan rate of 100 mV.s^{-1}**.**

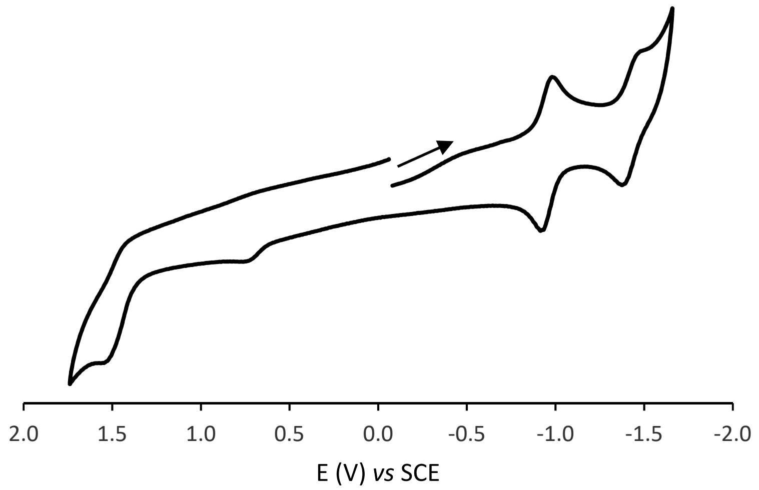

Figure S5. Cyclic voltammogram of ***3e*** in ACN + $TBAPF_6$ at scan rate of 100 $mV.s^{-1}$.

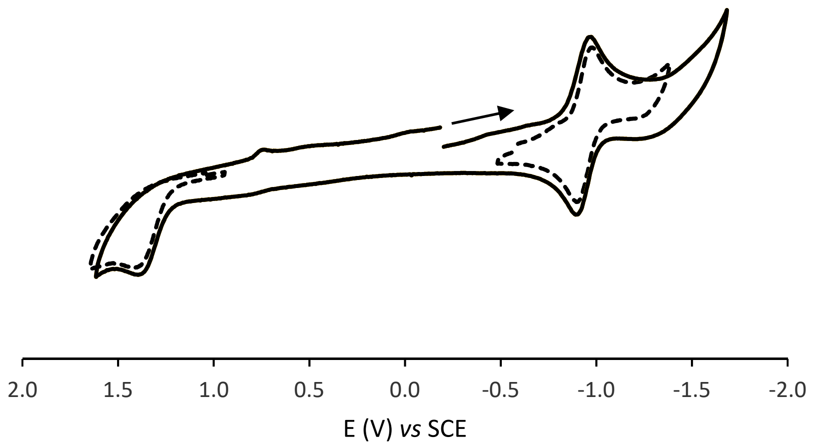

Figure S6. Cyclic voltammogram of ***3f*** in ACN + $TBAPF_6$ (black line) showing the isolated redox processes (dashed line) at scan rate of 100 $mV.s^{-1}$.

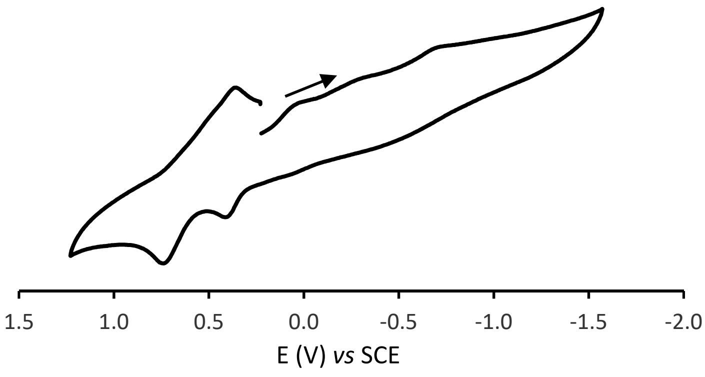

Figure S7. Cyclic voltammogram of ***4a*** in ACN + $TBAPF_6$ showing the ferrocene redox pair at scan rate of 100 $mV.s^{-1}$.

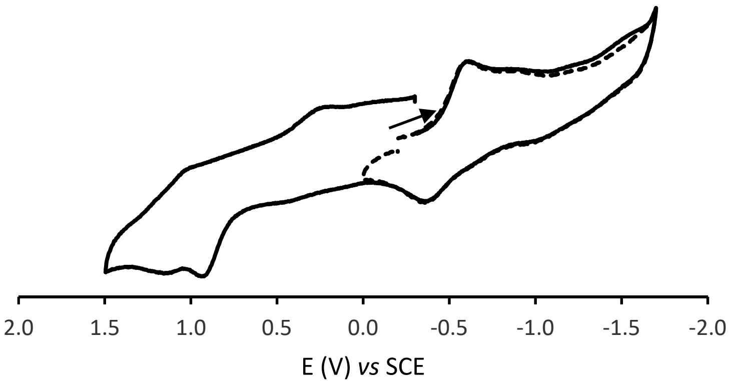

Figure S8. Cyclic voltammogram of ***4b*** in ACN + $TBAPF_6$ (black line) showing the isolated redox process (dashed line) at scan rate of 100 mV.s^{-1}.

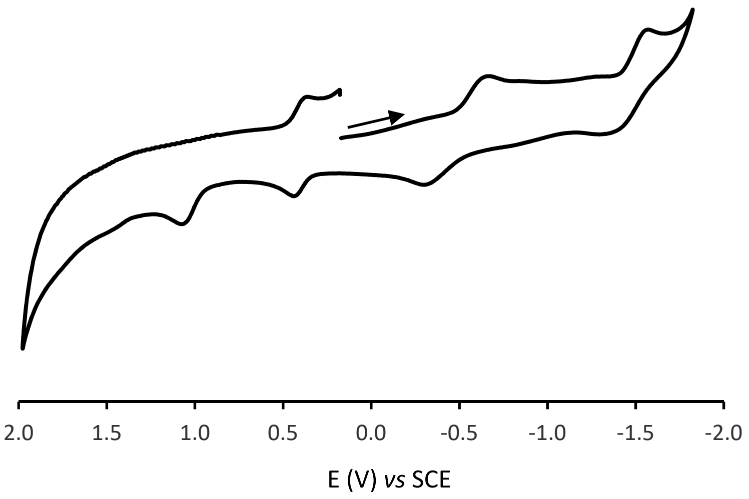

Figure S9. Cyclic voltammogram of ***4c*** in ACN + $TBAPF_6$ showing the ferrocene redox pair at scan rate of 100 mV.s^{-1}.

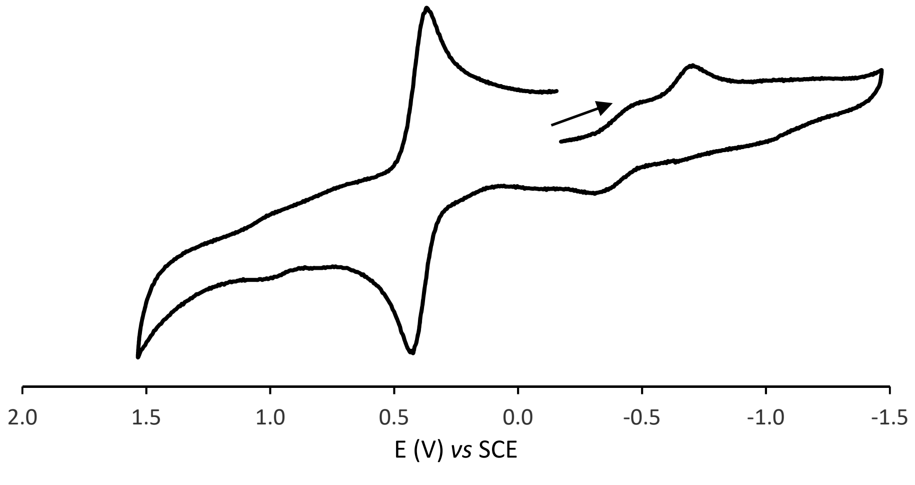

Figure S10. Cyclic voltammogram of ***4d*** in ACN + $TBAPF_6$ showing the ferrocene redox pair at scan rate of 100 mV.s^{-1}.

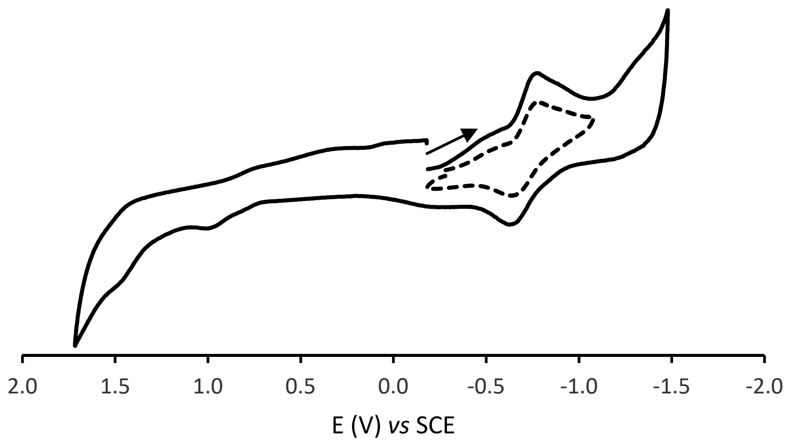

Figure S11. Cyclic voltammogram of ***4e*** in ACN + $TBAPF_6$ (black line) showing the isolated redox process (dashed line) at scan rate of 100 $mV.s^{-1}$.

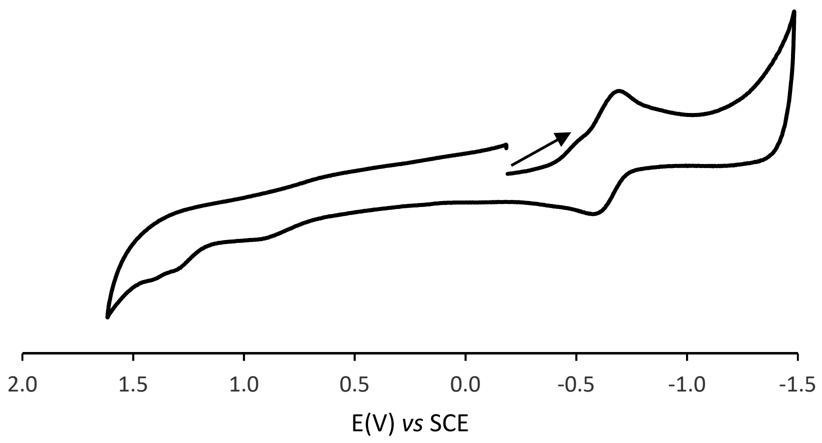

Figure S12. Cyclic voltammogram of ***4f*** in ACN + $TBAPF_6$ at scan rate of 100 $mV.s^{-1}$.

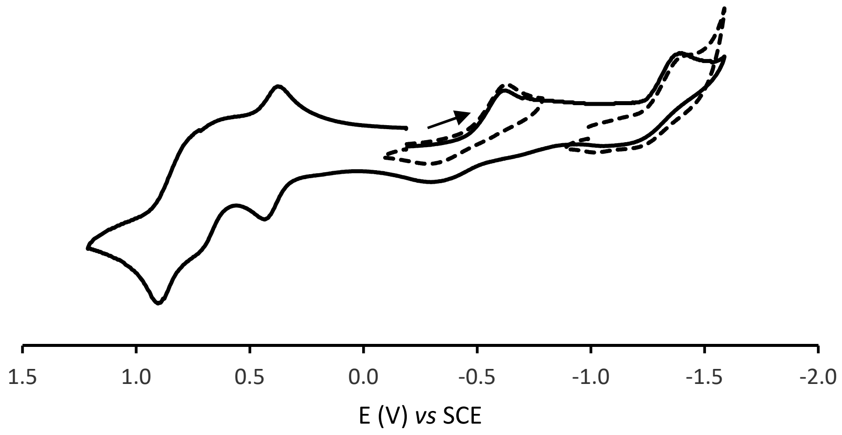

Figure S13. Cyclic voltammogram of ***4g*** in ACN + $TBAPF_6$ showing the ferrocene redox pair (black line) and the isolated redox processes (dashed line) at scan rate of 100 $mV.s^{-1}$.

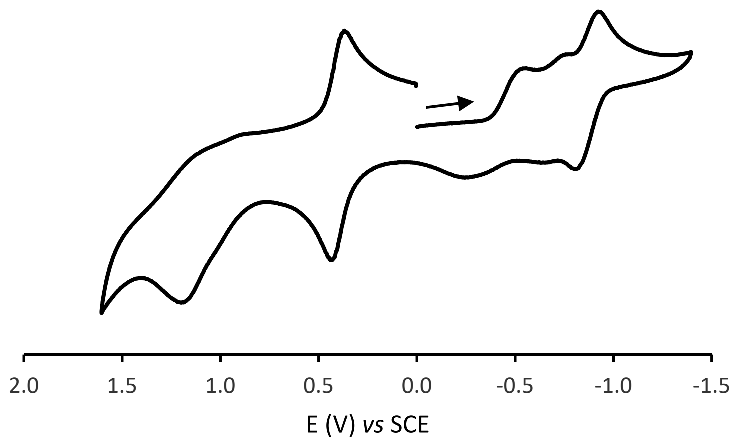

Figure S14. Cyclic voltammogram of ***4h*** in ACN + $TBAPF_6$ showing the ferrocene redox pair (black line) and the isolated redox processes (dashed line) at scan rate of 100 $mV.s^{-1}$.

3. *In silico* analysis

Table S3. Lipinski's parameters for compounds ***1*** and ***3a-f*** estimated using Molinspiration.

Compound	Lipinski's parameters							ABS%[1]
	M (g/mol)	mi LogP	TPSA	n_{OH}	n_{OHNH}	n_{rot}	$n_{violations}$	
1	158.16	1.67	34.14	2	0	0	0	98.83
3a	264.28	2.66	72.19	4	3	2	0	84.55
3b	265.27	3.11	66.4	4	2	2	0	92.53
3c	279.3	3.64	55.4	4	1	3	0	97.78
3d	274.28	3.34	69.96	4	1	2	0	97.60
3e	229.28	2.89	46.17	3	1	4	0	97.42
3f	229.28	2.45	37.38	3	0	3	0	99.37

Table S4. Lipinski's parameters for compounds ***2*** and ***4a-h*** estimated using Molinspiration.

Compound	Lipinski's parameters							ABS%[1]
	M (g/mol)	mi LogP	TPSA	n_{OH}	n_{OHNH}	n_{rot}	$n_{violations}$	
2	158.16	1.67	34.14	2	0	0	0	98.83
4a	264.28	2.66	72.19	4	3	2	0	84.55
4b	265.27	3.11	66.4	4	2	2	0	92.53
4c	279.30	3.64	55.4	4	1	3	0	97.78
4d	274.28	3.34	69.96	4	1	2	0	97.60
4g	340.38	5.58	91.99	4	2	2	0	97.18
4h	265.27	3.32	66.4	4	2	2	0	92.53
4e	229.28	2.89	46.17	3	1	4	0	97.42
4f	229.28	2.45	37.38	3	0	3	0	99.37

$$\mathbf{ABS\%} = \mathbf{100} \times \left[\mathbf{exp}\left(-\mathbf{10}^{\mathbf{0.506-0.0754\times n_{OH}-0.141\times n_{OHNH}+0.00184\times MM}}\right)\right]$$

Table S5. Bioactivity scores compounds ***1***, ***2***, ***3a-f*** and ***4a-h*** estimated using Molinspiration.

Compound	GPCR ligand	Ion channel modulator	Kinase inhibitor	Nuclear receptor ligand	Protease inhibitor	Enzyme inhibitor
1	-0.94	-0.46	-0.77	-1	-1.1	-0.34
3a	-0.11	-0.25	**0.46**	-0.6	-0.3	**0.20**
3b	-0.12	-0.28	**0.40**	-0.12	-0.4	**-0.14**
3c	-0.19	-0.41	**0.32**	-0.26	-0.42	**0.02**
3d	-0.11	-0.32	**0.47**	-0.13	-0.33	**0.12**
3e	-0.17	-0.32	-0.02	-0.37	-0.42	0.23
3f	-0.19	-0.36	-0.06	-0.46	-0.62	0.04
2	-0.87	-0.4	-0.76	-1.18	-0.91	-0.05
4a	0.01	0.01	**0.46**	-0.34	-0.06	**0.43**
4b	-0.01	-0.02	**0.39**	-0.11	-0.16	**0.37**
4c	-0.07	-0.16	**0.31**	-0.24	-0.20	**0.24**
4d	0.00	-0.07	**0.47**	-0.12	-0.10	**0.34**
4g	0.03	-0.05	**0.33**	-0.13	-0.01	**0.24**
4h	0.00	-0.32	**0.32**	-0.32	-0.25	**0.32**
4e	-0.12	-0.01	**-0.01**	-0.47	-0.23	**0.34**
4f	-0.16	-0.14	**-0.08**	-0.45	-0.38	**0.20**

A molecule having bioactivity score more than 0.00 is most likely to exhibit considerable biological activities, while values -0.50 to 0.00 are expected to be moderately active and if score is less than -0.50 it is presumed to be inactive.

4. *In vitro* cytotoxicity against human hepatocarcinoma cell line HepG2

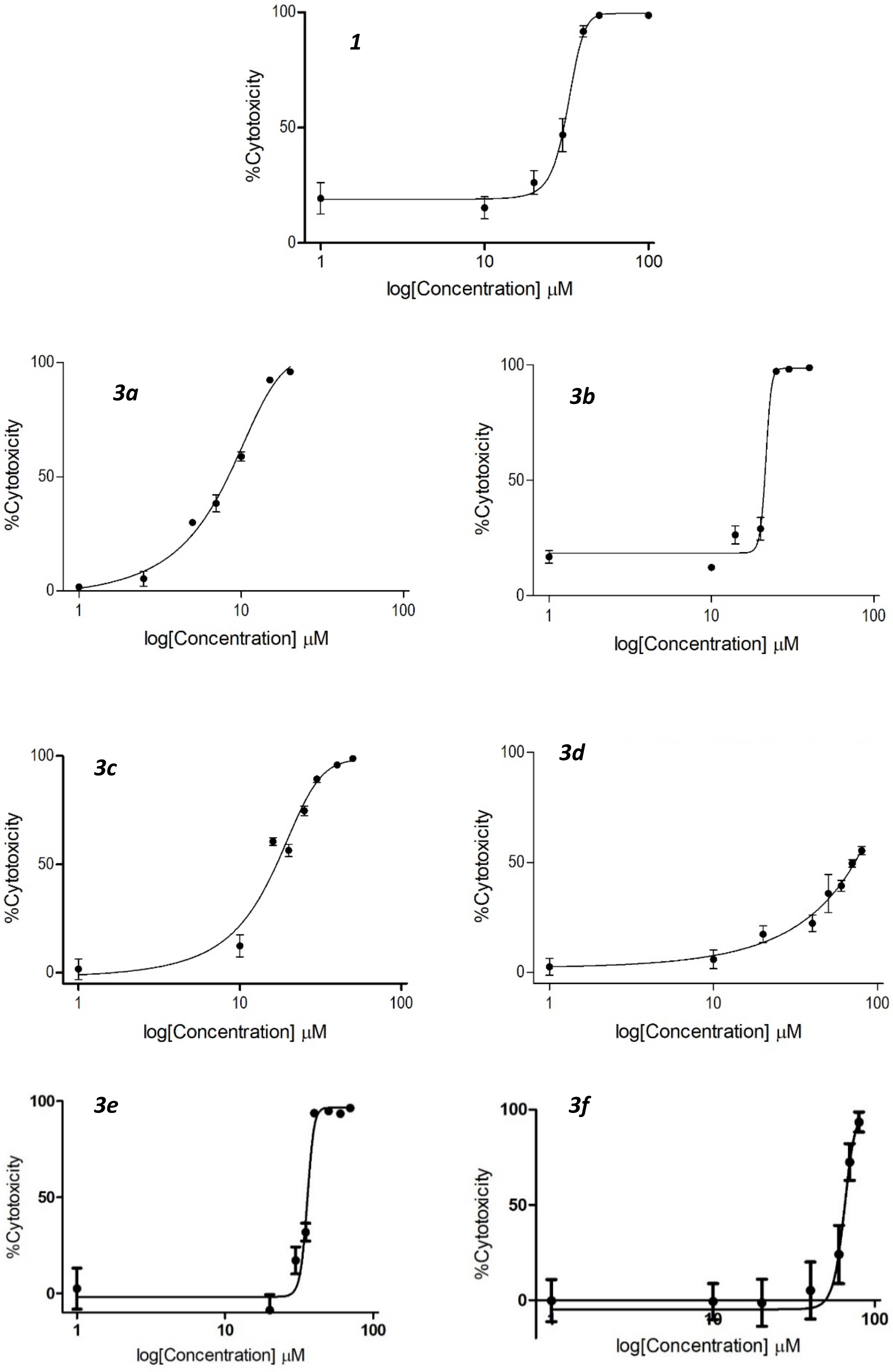

Figure S15. Cytotoxicity profiles of compounds ***1*** and ***3a-f*** in HepG2 human cell line.

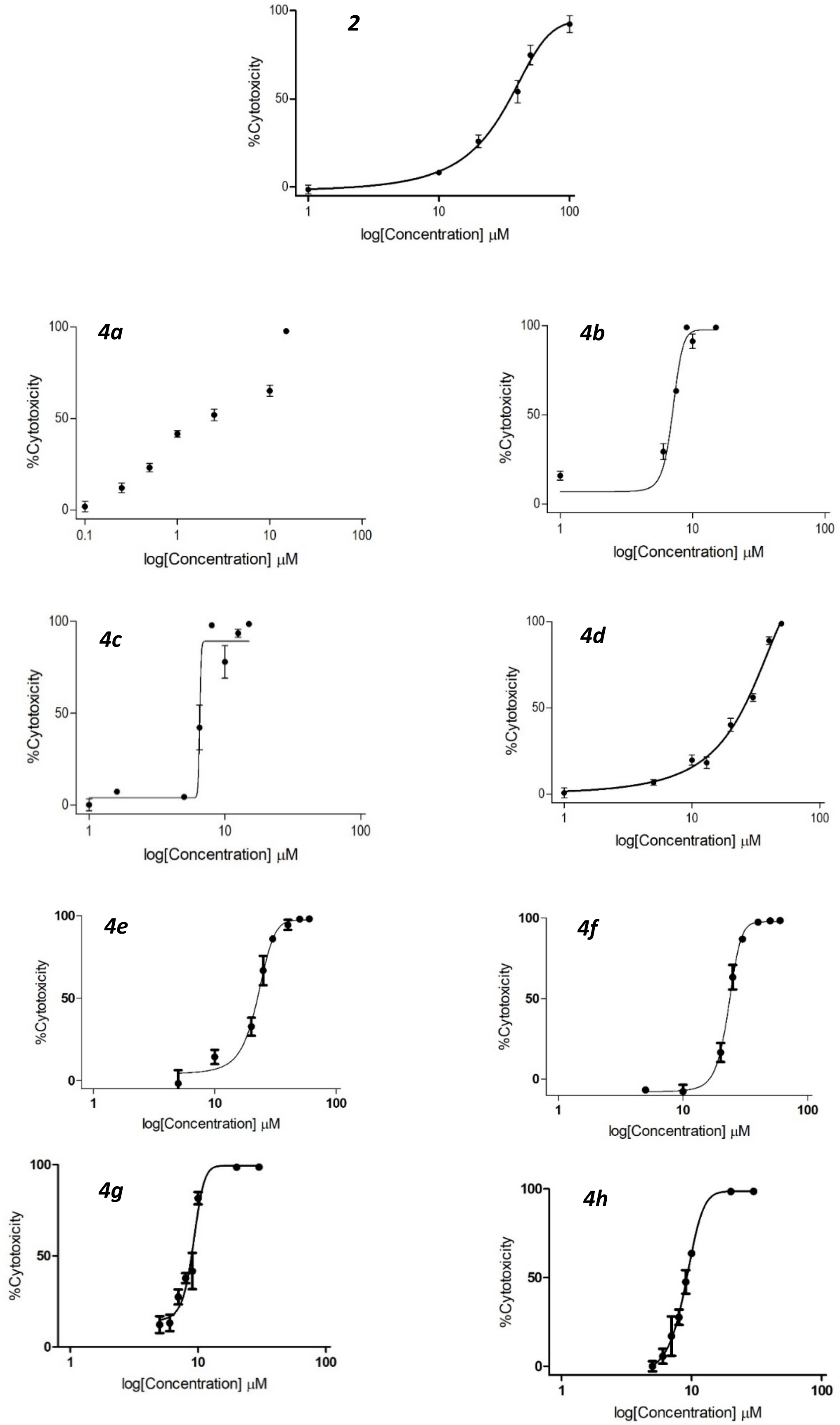

Figure S16. Cytotoxicity profiles of compound ***2*** and ***4a-h*** in HepG2 human cell line.

Table S6. The EC_{50} values for the cytotoxicity of compounds ***1***, ***2***, ***3a-f***, ***4a-h*** and reference inhibitor doxorubicin against HepG2 cell line.

Compound	EC_{50} (µM±SD)
1	32.2 ± 1.4
3a	8.7 ± 1.3
3b	21.4 ± 3.9
3c	13.0 ± 1.8
3d	79.4 ± 1.4
3e	35.8 ± 1.5
3f	64.7 ± 1.4
2	25.6 ± 1.1
4a	2.7 ± 1.0
4b	7.1 ± 1.8
4c	6.5 ± 1.4
4d	26.8 ± 1.1
4g	9.2 ± 2.3
4h	9.1 ± 3.3
4e	26.8 ± 1.1
4f	23.1 ± 1.7
Doxorubicin	1.42 ± 1.0

All EC_{50} values are expressed as the mean ± SD of triplicate determinations.

5. Inhibition of acetylcholinesterase activity

Table S7. AChE $Inhibiton_{50\mu M}$ values for compounds ***1***, ***2***, ***3a-f***, ***4a-h***.

Compound	$Inhibiton_{50\mu M}$ (µM±SD)
1	50.7 ± 2.3
3a	23.4 ± 2.5
3b	16.4 ± 2.0
3c	25.9 ± 3.0
3d	25.8 ± 3.0
3e	48.3 ± 1.6
3f	47.9 ± 2.5
2	34.9 ± 5.2
4a	51.7 ± 2.1
4b	74.1 ± 1.1
4c	45.8 ± 2.9
4d	41.6 ± 2.4
4g	75.3 ± 1.3
4h	85.3 ± 1.3
4e	62.4 ± 1.5
4f	54.71 ± 2.0

All $Inhibiton_{50\mu M}$ values are expressed as the mean ± SD of triplicate determinations.

6. Molecular docking studies

Table S8. Molecular docking results of ***3b***.

AA	Atom	Interaction type	Distance (Å)
Phe 295	O=C (Quinone ring)	N-H···O(O=C)[a]	2,14
Val 294	O=C (Quinone ring)	C-H···O(O=C)	2,70
Trp 286	C=C (Ar NQ ring)	π-π stacking	5,09
Trp 286	Ar (NQ ring)	π-π stacking	3,90
Trp 286	Ar (Quinone ring)	π-π stacking	5,41
Tyr 341	Ar (Quinone ring)	π-π stacking	4,60
Tyr 341	Ar (NQ ring)	π-π stacking	3,93

[a] Conventional Hydrogen Bond

Table S9. Molecular docking results of ***4b***.

AA	Atom	Interaction type	Distance (Å)
Gly 120	O-C (Ar ring)	N-H···O(O-C)	2,69
Tyr 124	O=C (Quinone ring)	O-H···O(O=C)	2,69
Tyr 133	O-C (Ar ring)	O-H···O(O-C)	2,35
Glu 202	H-O (Ar ring)	O···H-O(O-C)	1,89
Trp 86	Ar ring	π-π stacking	4,61
Trp 86	Ar ring	π-π stacking	3,76
Tyr 337	Ar (NQ ring)	π-π stacking	4,06
Tyr 337	Ar (Quinone ring)	π-π stacking	5,36
Tyr 341	Ar (NQ ring)	π-π stacking	4,83
Tyr 341	Ar (Quinone ring)	π-π stacking	5,55

[a] Conventional Hydrogen Bond

Table S10. Molecular docking results of ***4h***.

AA	Atom	Interaction type	Distance (Å)
Tyr 133	O=C (Quinone ring)	O-H···O(O=C)	3,46
Gly 126	O=C (Quinone ring)	O-H···O(O=C)	4,70
Tyr 337	H-N (Ar ring)	O···H-N	3,09
Tyr 341	H-O (Ar ring)	O···H-O(O-C)	3,26
Tyr 341	Ar ring	π-π stacking	4,25
Tyr 337	Ar ring	π-π stacking	4,69
Trp 86	Ar (NQ ring)	π-π stacking	4,15
Trp 86	Ar (Quinone ring)	π-π stacking	4,24

[a] Conventional Hydrogen Bond

7. 1D and 2D NMR spectra

Compound *3a*:

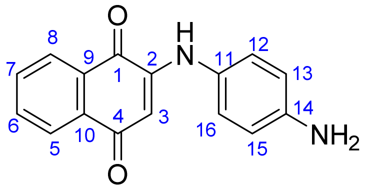

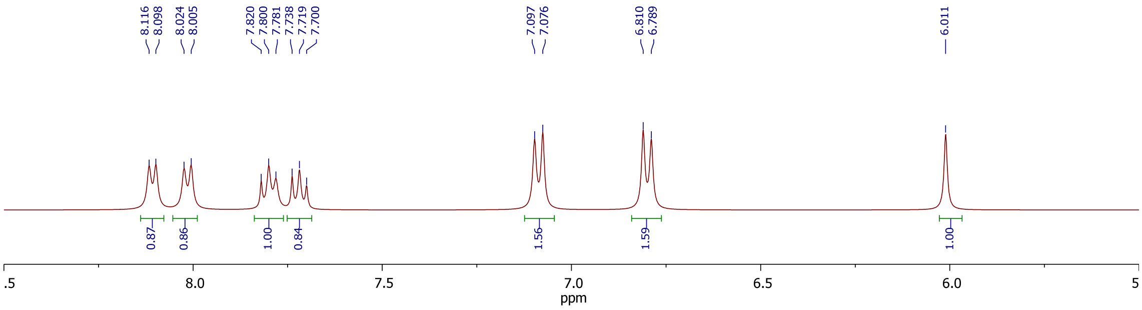

Figure S17. ^{1}H-NMR spectrum of compound ***3a*** in MeOD-d_4

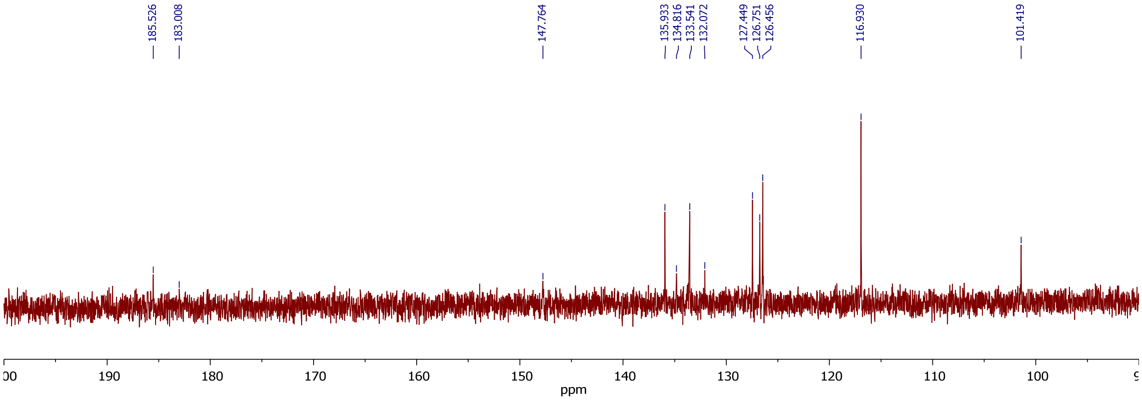

Figure S18. ^{13}C-NMR spectrum of compound ***3a*** in MeOD-d_4

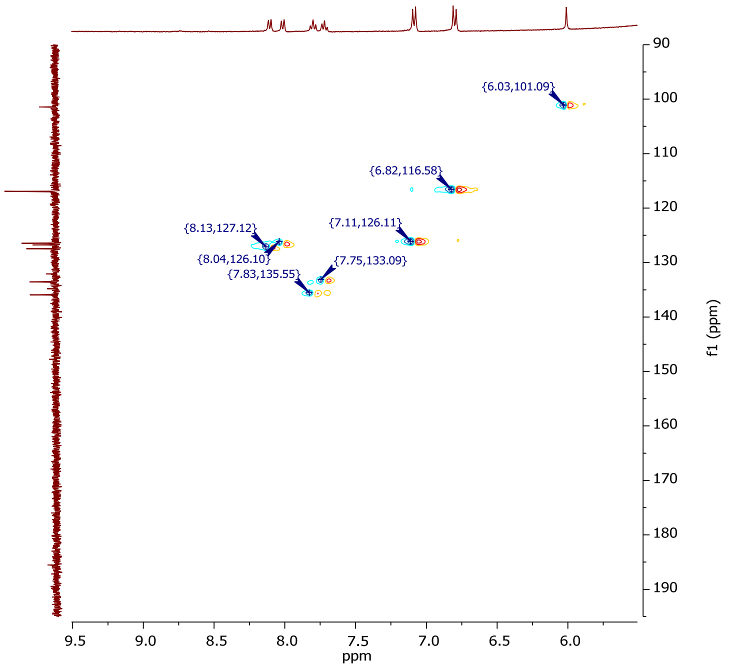

Figure S19. HSQC-NMR spectrum of compound ***3a*** in MeOD-d_4

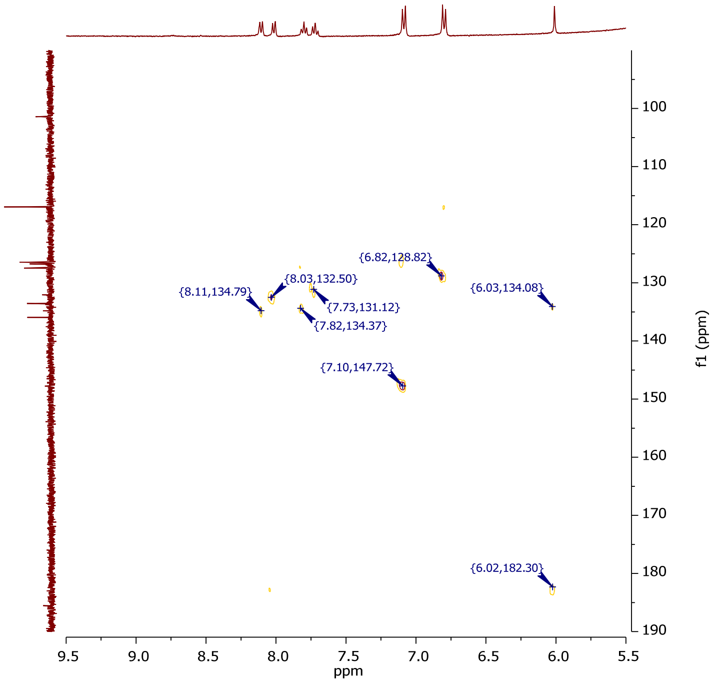

Figure S20. HMBC-NMR spectrum of compound ***3a*** in MeOD-d_4

Compound *3b*:

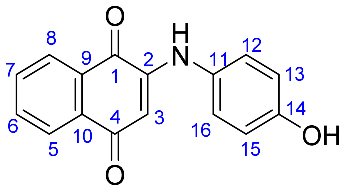

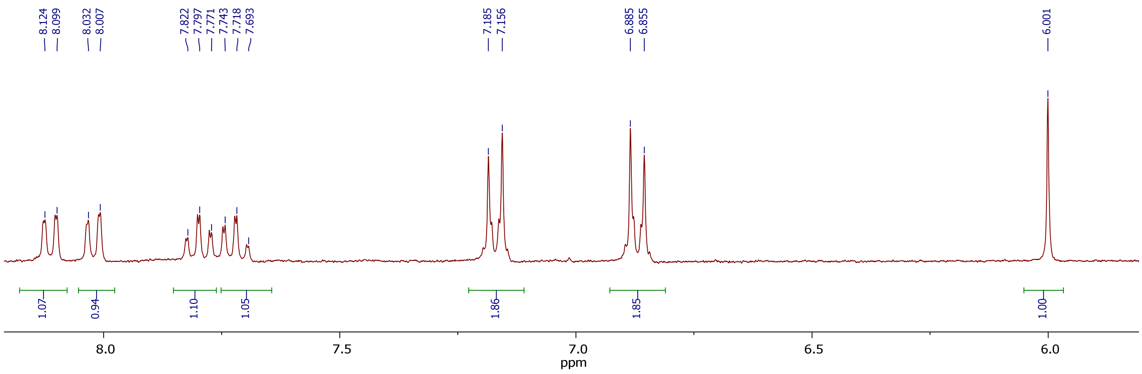

Figure S21. ^{1}H-NMR spectrum of compound ***3b*** in MeOD-d_4

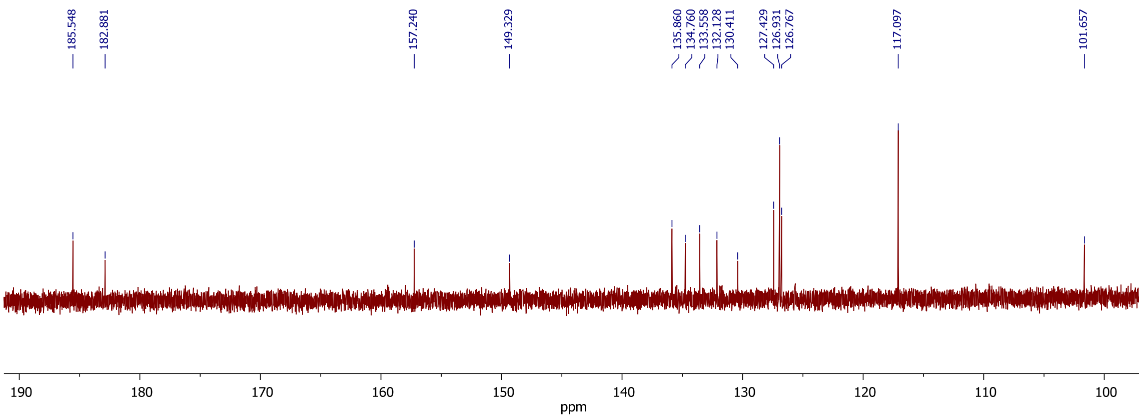

Figure S22. ^{13}C-NMR spectrum of compound ***3b*** in MeOD-d_4

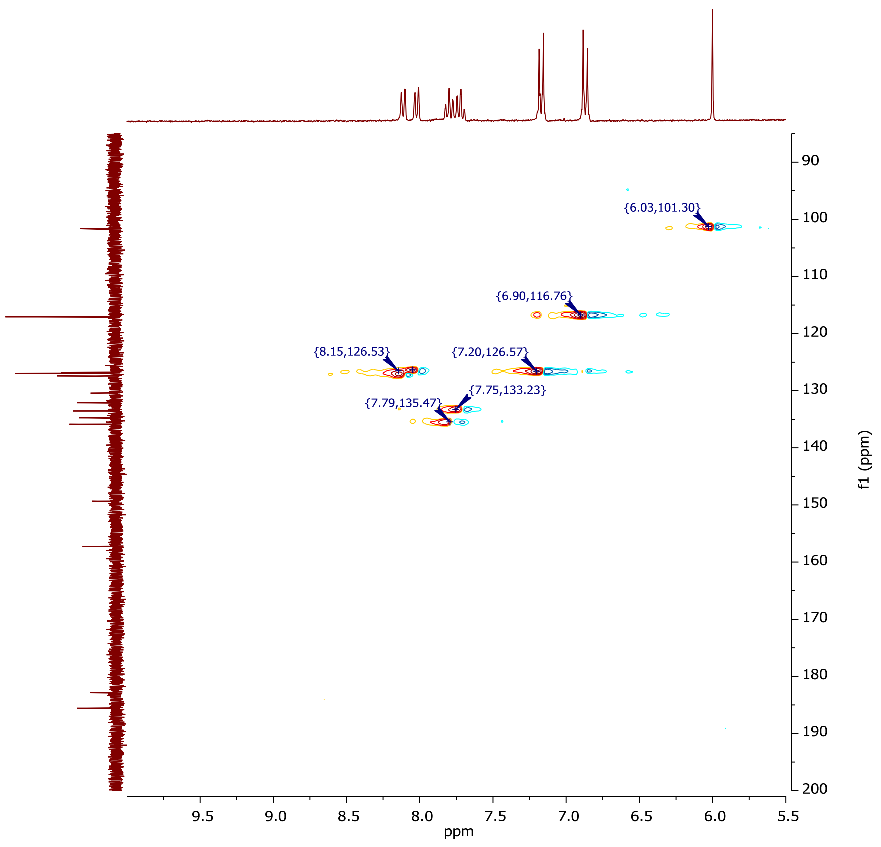

Figure S23. HSQC-NMR spectrum of compound ***3b*** in MeOD-d_4

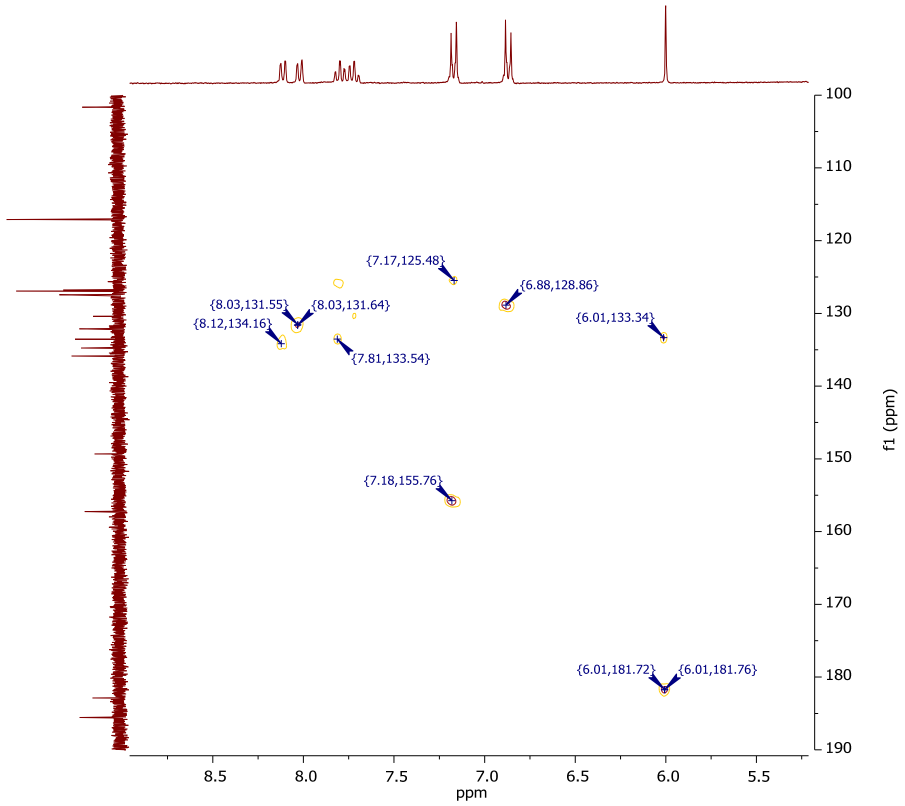

Figure S24. HMBC-NMR spectrum of compound ***3b*** in MeOD-d_4

Compound *3c*:

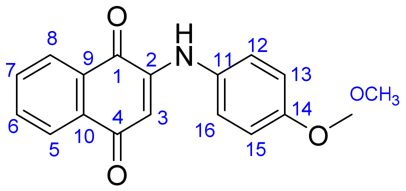

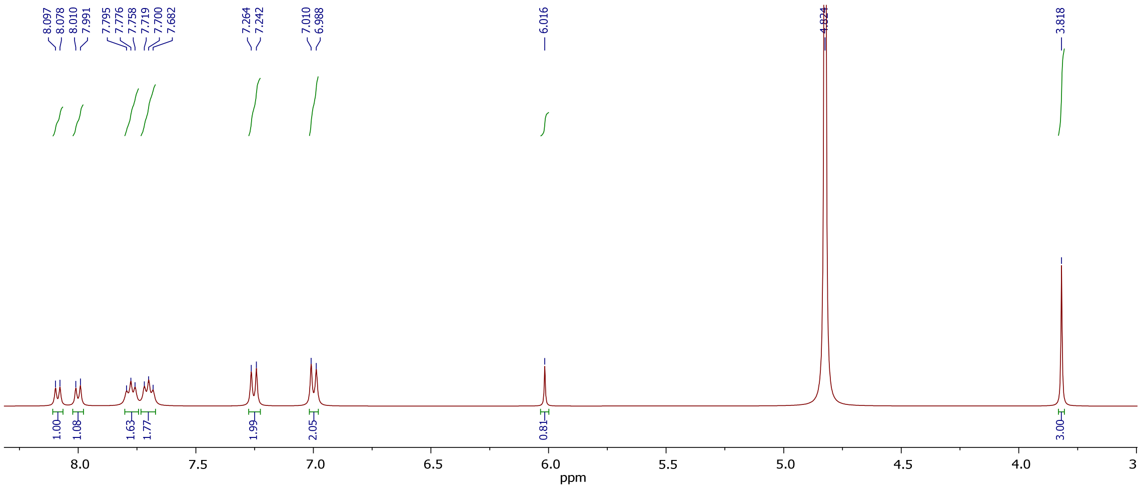

Figure S25. ^{1}H-NMR spectrum of compound ***3c*** in MeOD-d_4

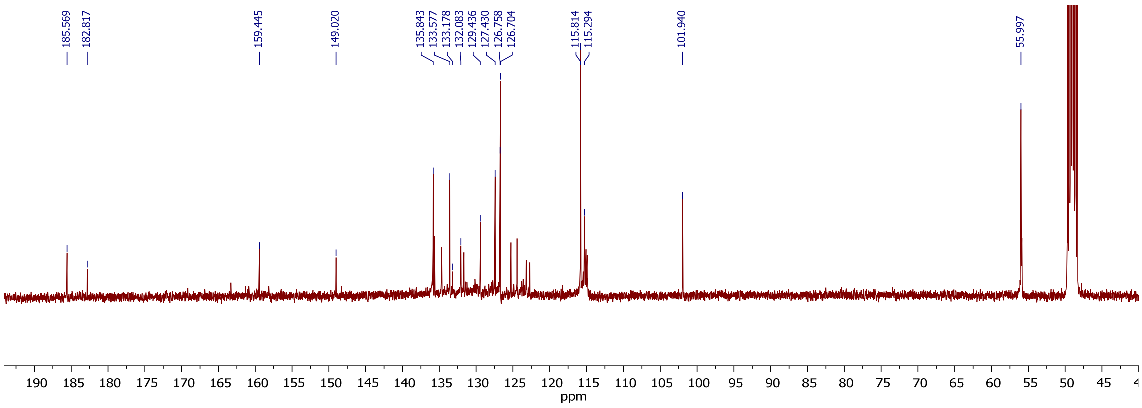

Figure S26. ^{13}C-NMR spectrum of compound ***3c*** in MeOD-d_4

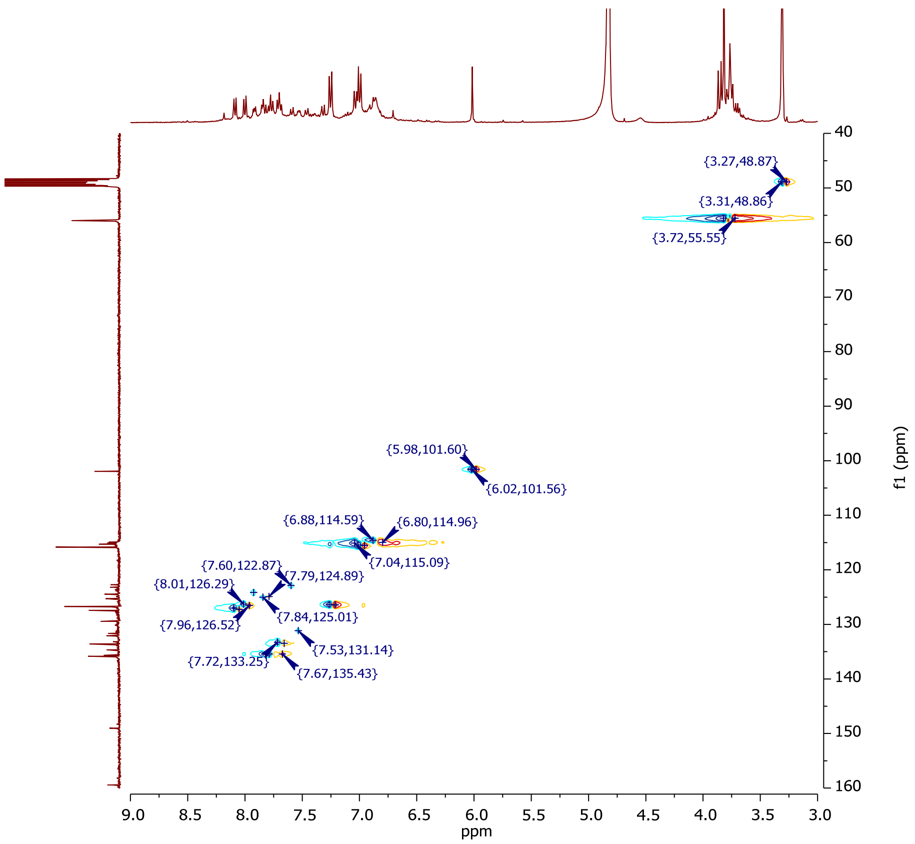

Figure S27. HSQC-NMR spectrum of compound ***3c*** in MeOD-d_4

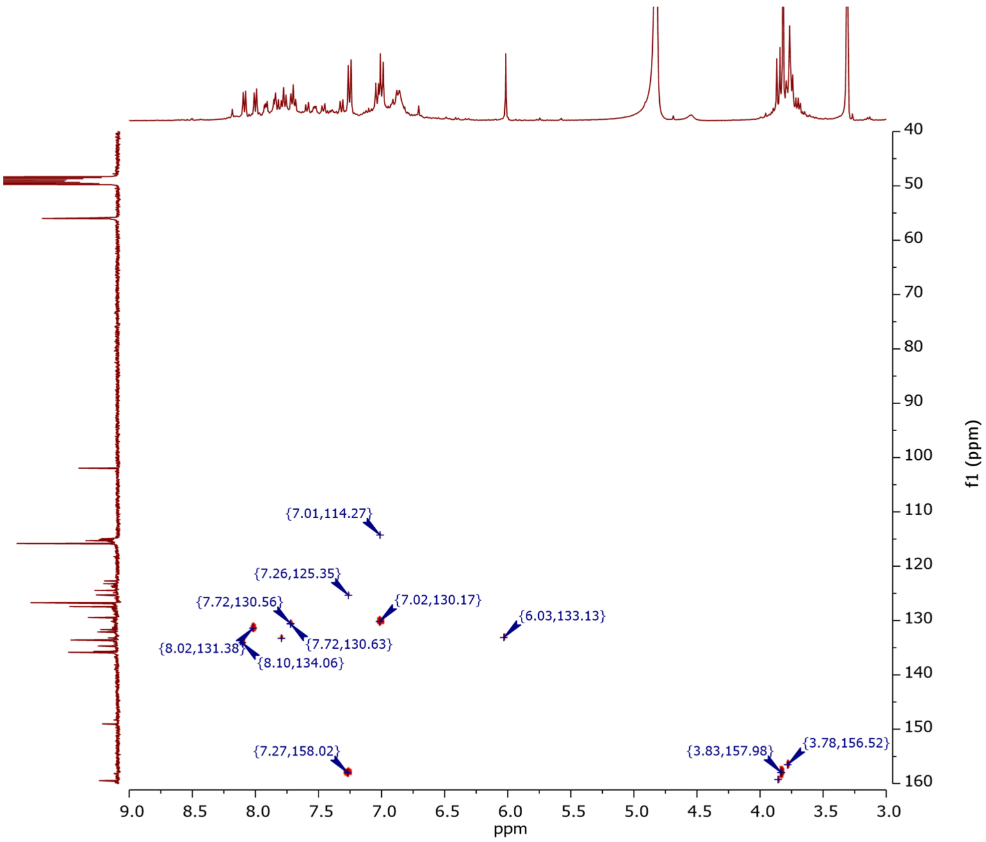

Figure S28. HMBC-NMR spectrum of compound ***3c*** in MeOD-d_4

Compound 3d:

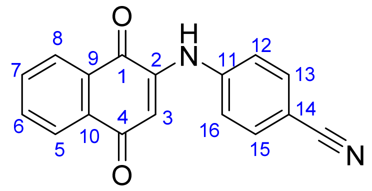

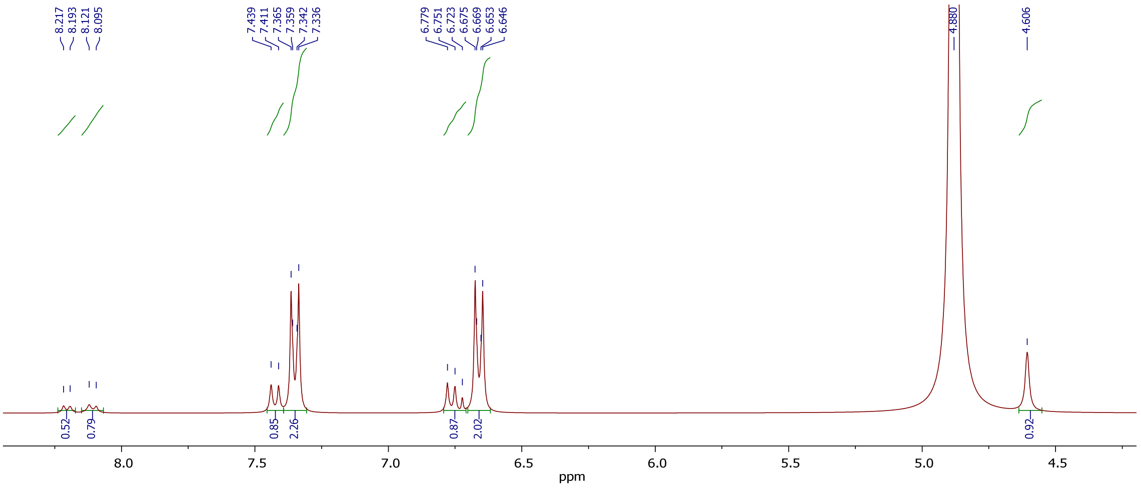

Figure S29. ^{1}H-NMR spectrum of compound ***3d*** in MeOD-d_4

Compound 3e:

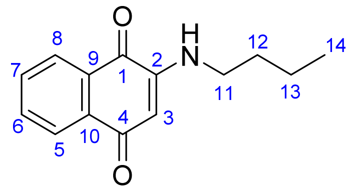

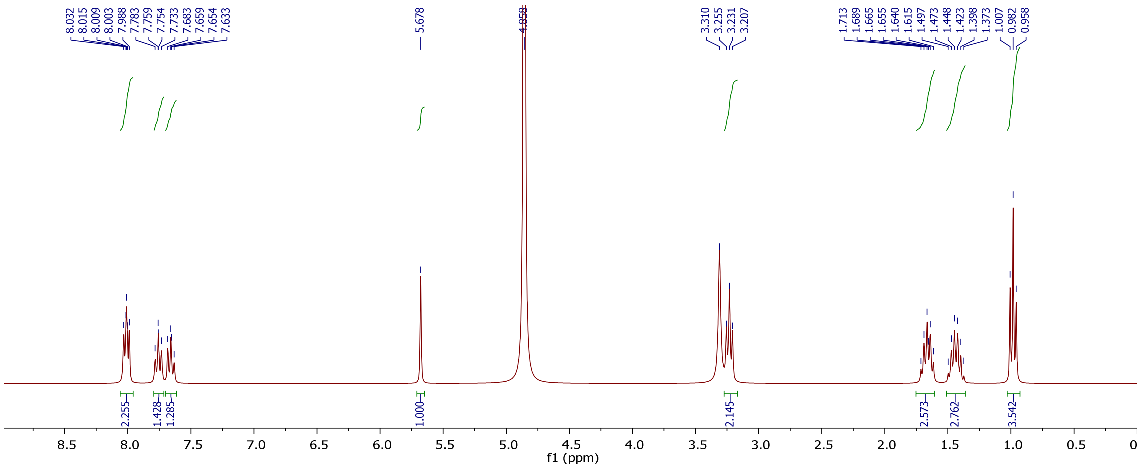

Figure S30. ^{1}H-NMR spectrum of compound ***3e*** in MeOD-d_4

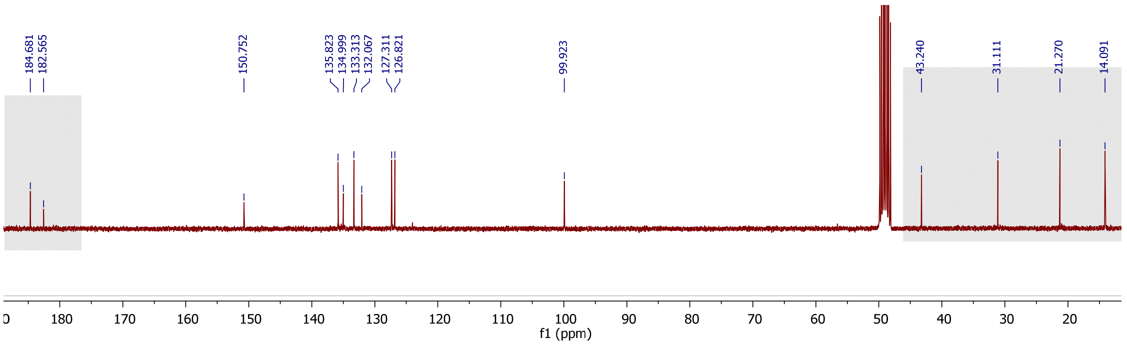

Figure S31. ^{13}C-NMR spectrum of compound ***3e*** in MeOD-d_4

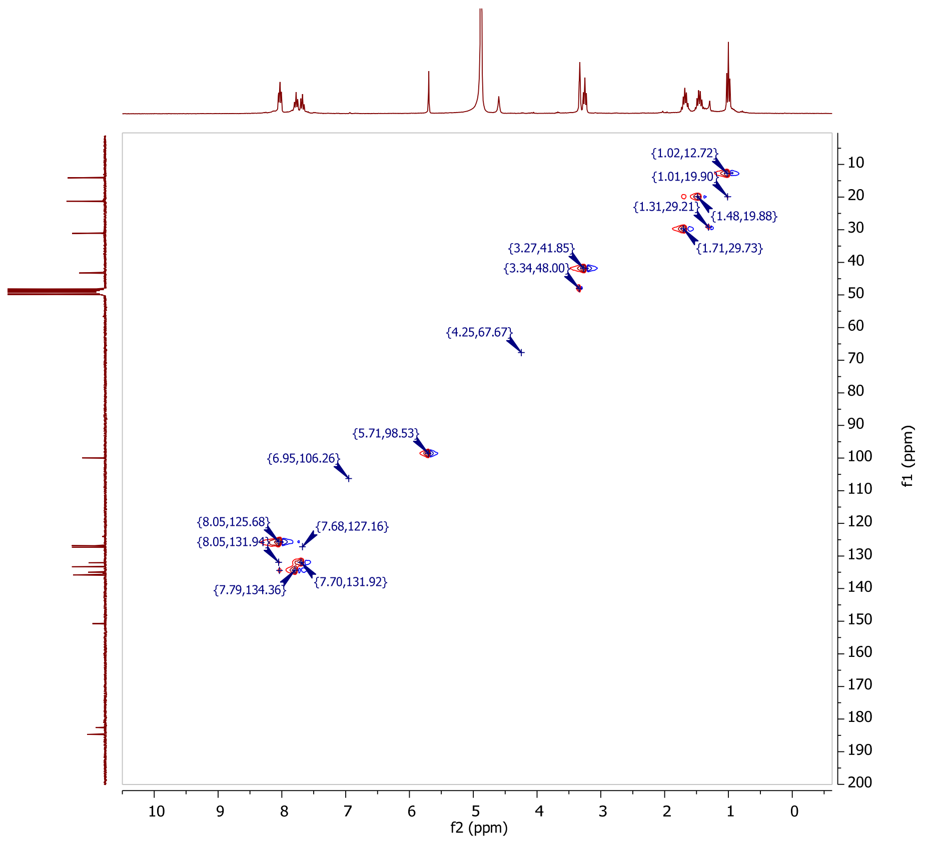

Figure S32. HSQC-NMR spectrum of compound ***3e*** in MeOD-d_4

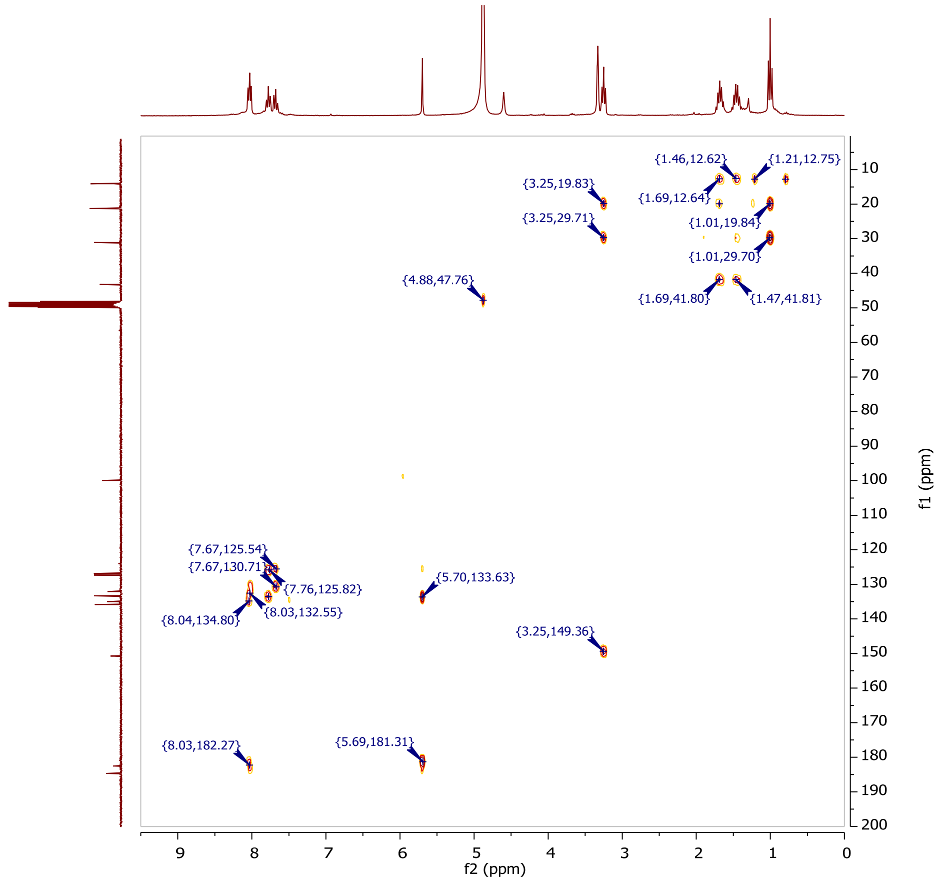

Figure S33. HMBC-NMR spectrum of compound ***3e*** in MeOD-d_4

Compound 3f:

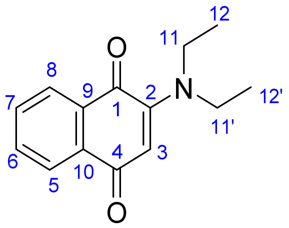

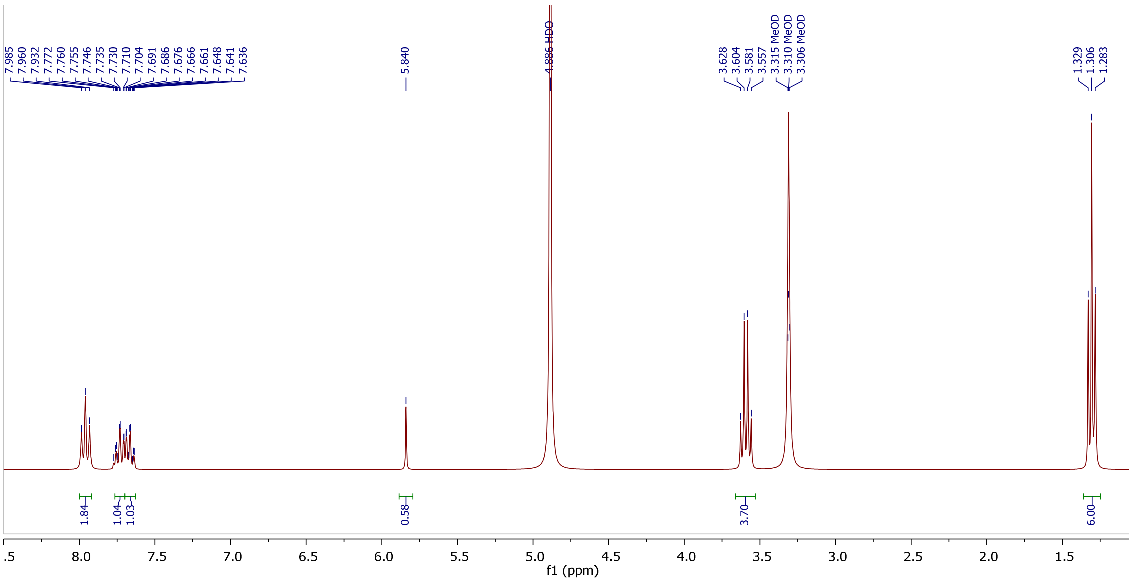

Figure S34. ^{1}H-NMR spectrum of compound ***3f*** in MeOD-d_4

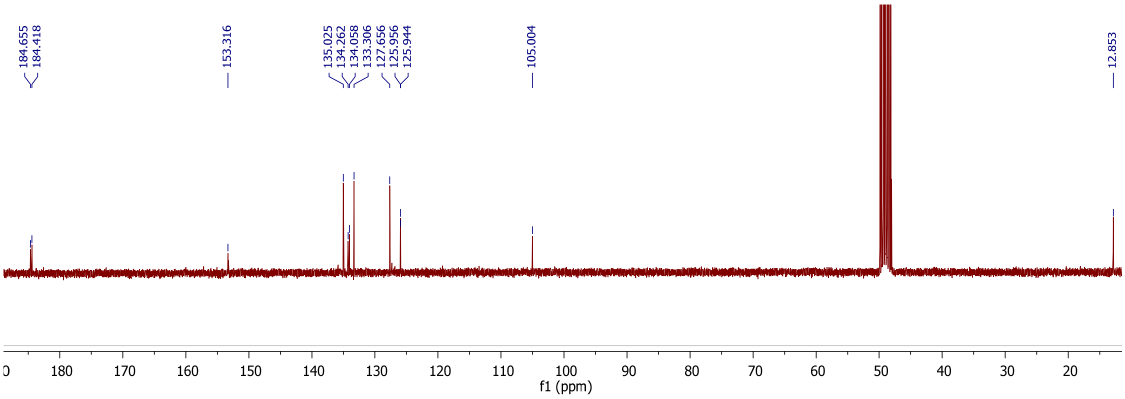

Figure S35. ^{13}C-NMR spectrum of compound ***3f*** in MeOD-d_4

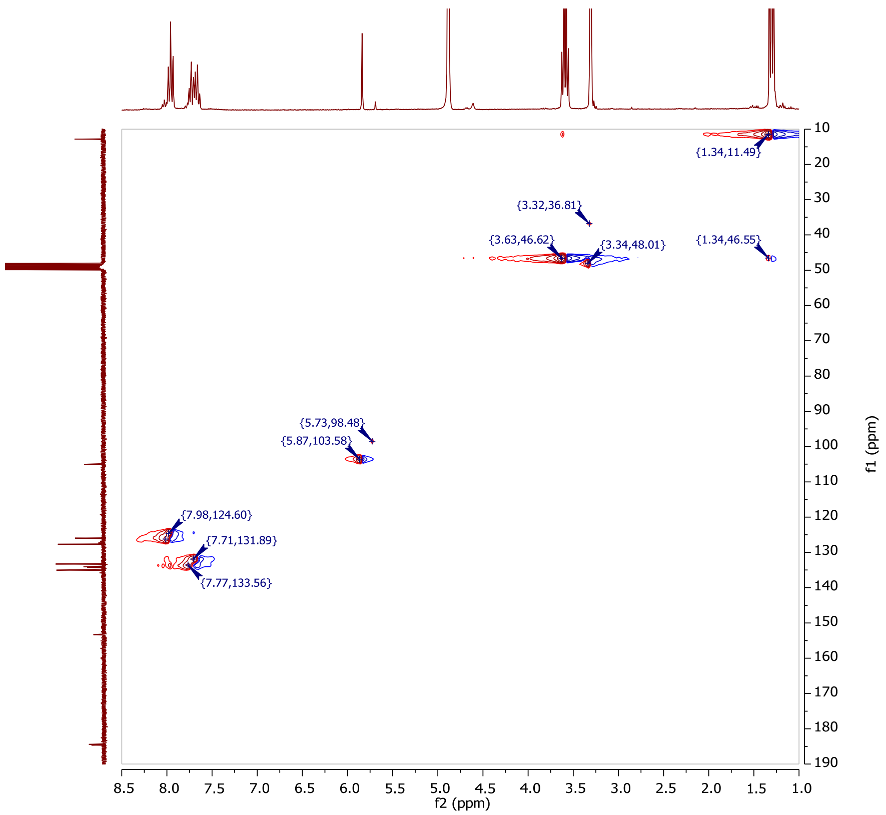

Figure S36. HSQC-NMR spectrum of compound ***3f*** in MeOD-d_4

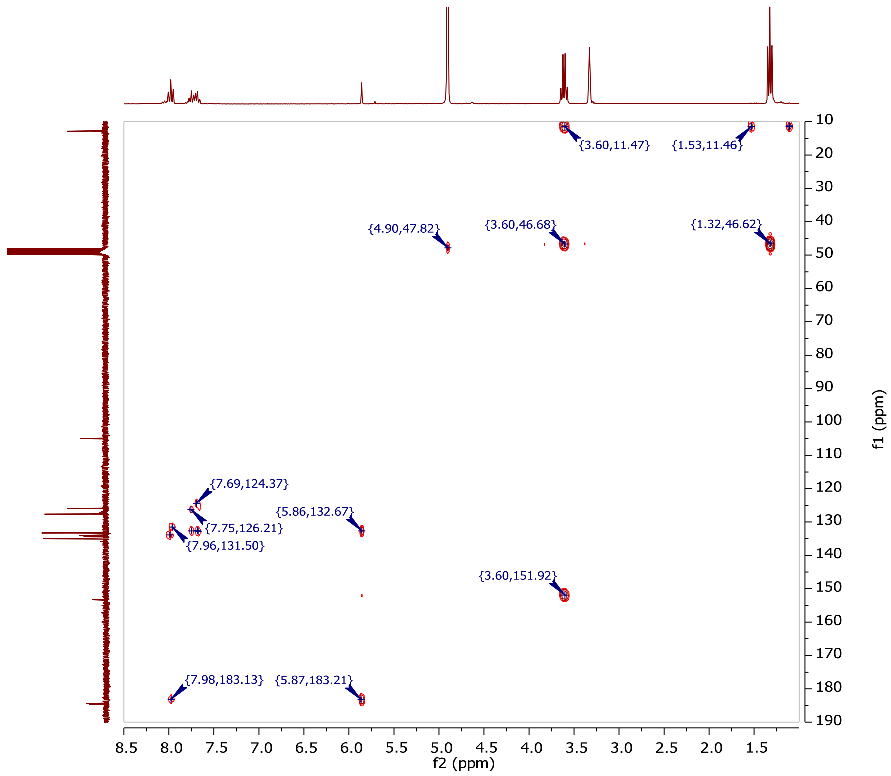

Figure S37. HMBC-NMR spectrum of compound ***3f*** in MeOD-d_4

Compound 4e:

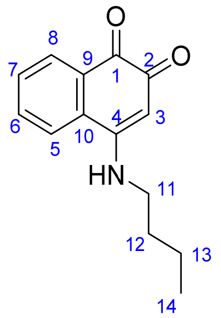

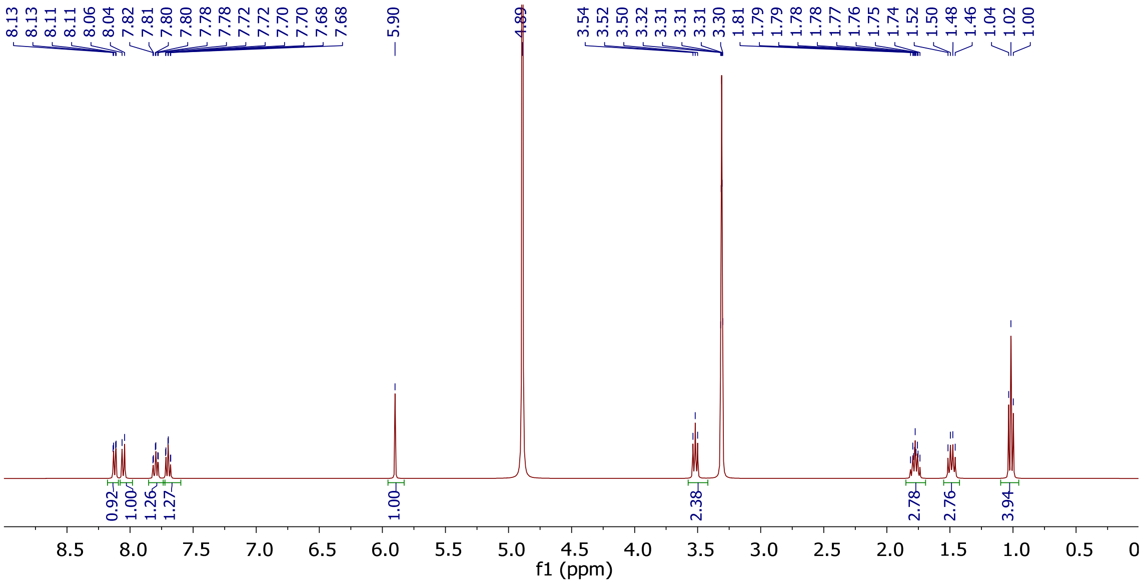

Figure S38. ^{1}H-NMR spectrum of compound ***4e*** in MeOD-d_4

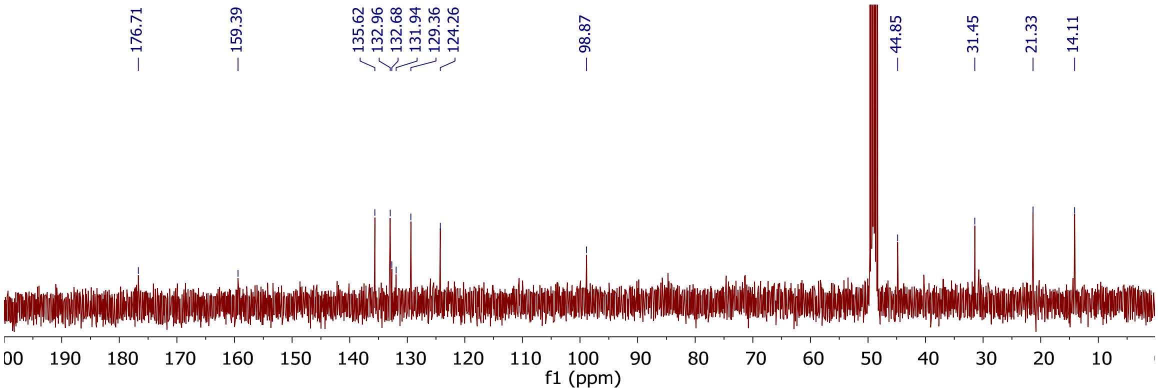

Figure S39. ^{13}C-NMR spectrum of compound ***4e*** in MeOD-d_4

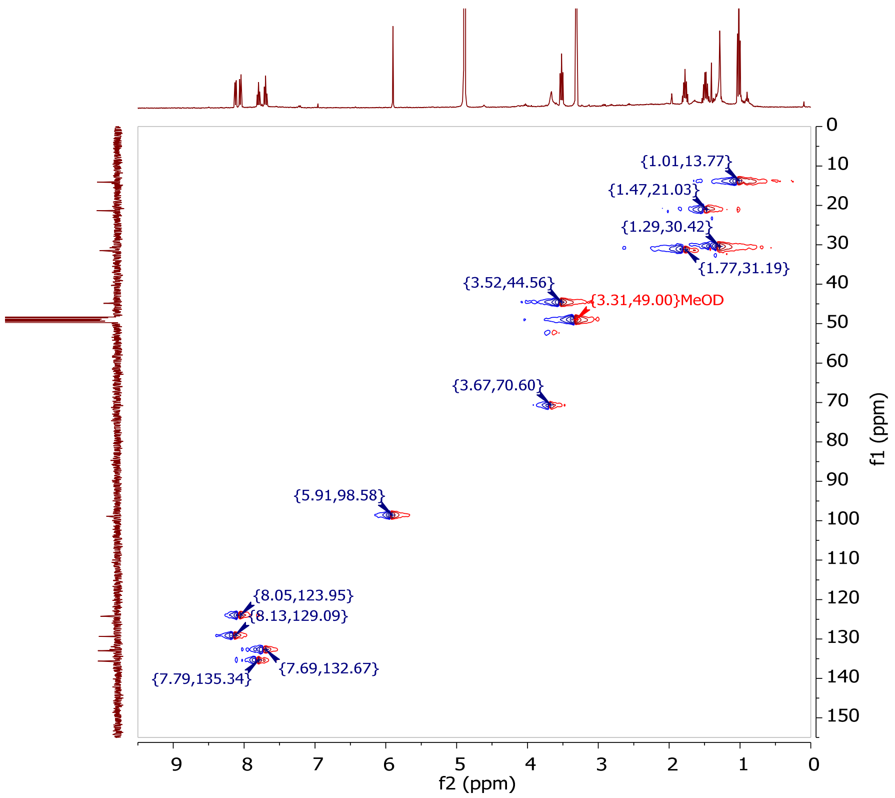

Figure S40. HSQC-NMR spectrum of compound ***4e*** in MeOD-d_4

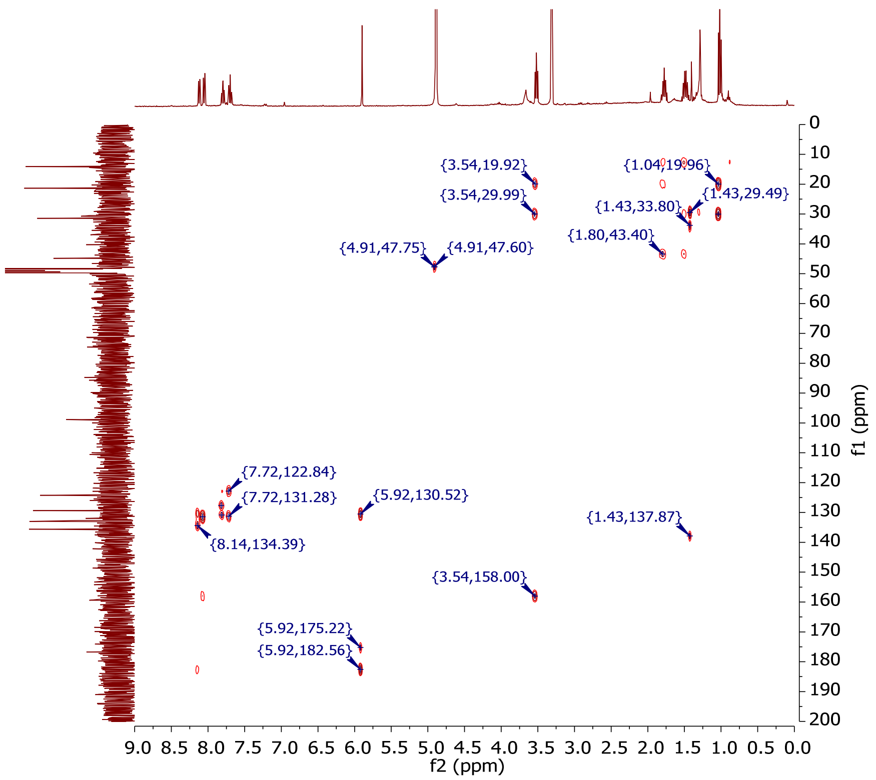

Figure S41. HMBC-NMR spectrum of compound ***4e*** in MeOD-d_4

Compound 4f:

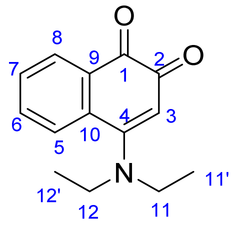

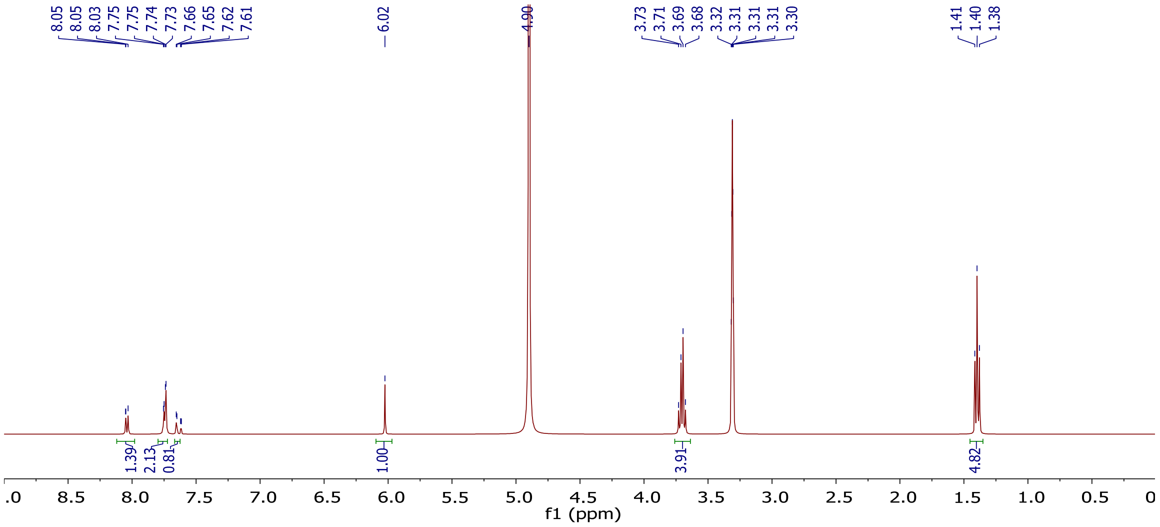

Figure 1S42. ^{1}H-NMR spectrum of compound ***4e*** in MeOD-d_4

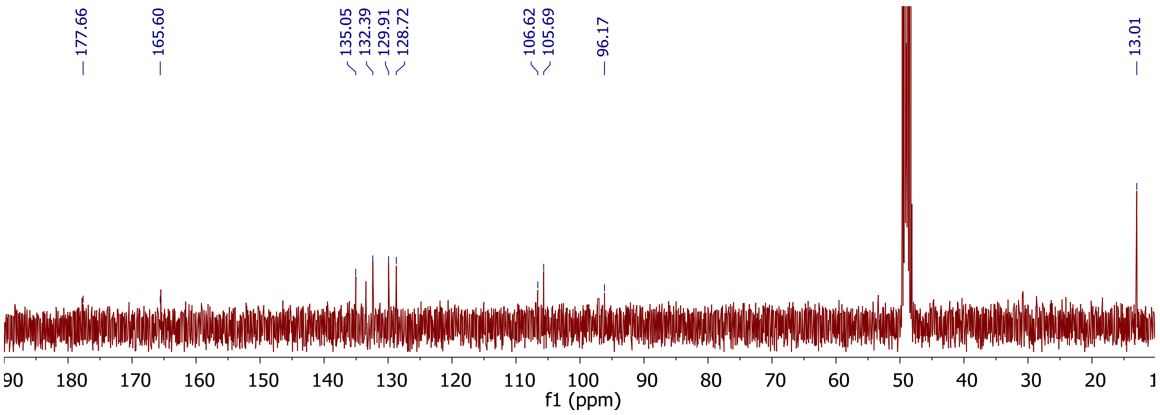

Figure S43. ^{13}C-NMR spectrum of compound ***4e*** in MeOD-d_4

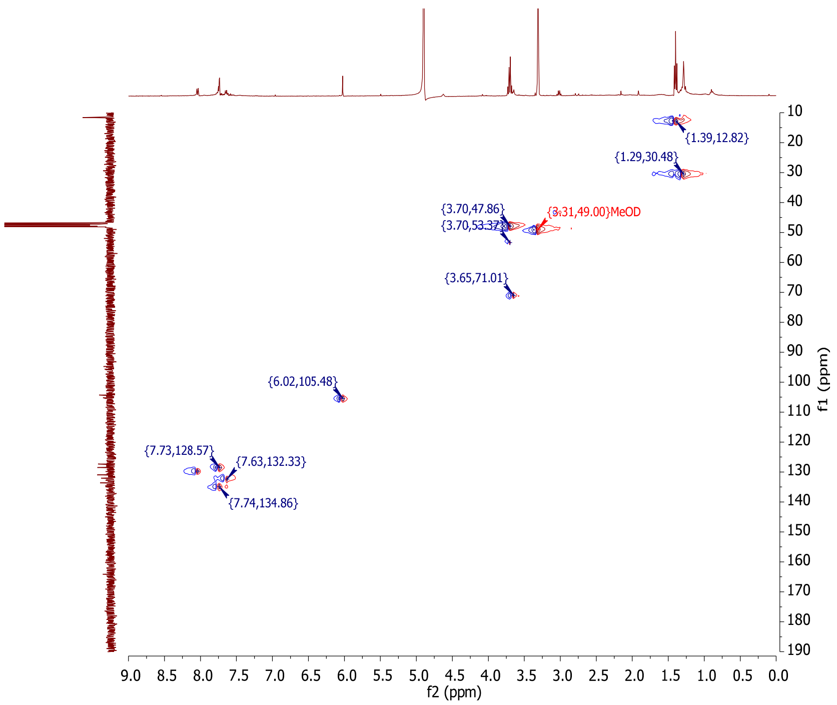

Figure S44. HSQC-NMR spectrum of compound ***4e*** in MeOD-d_4

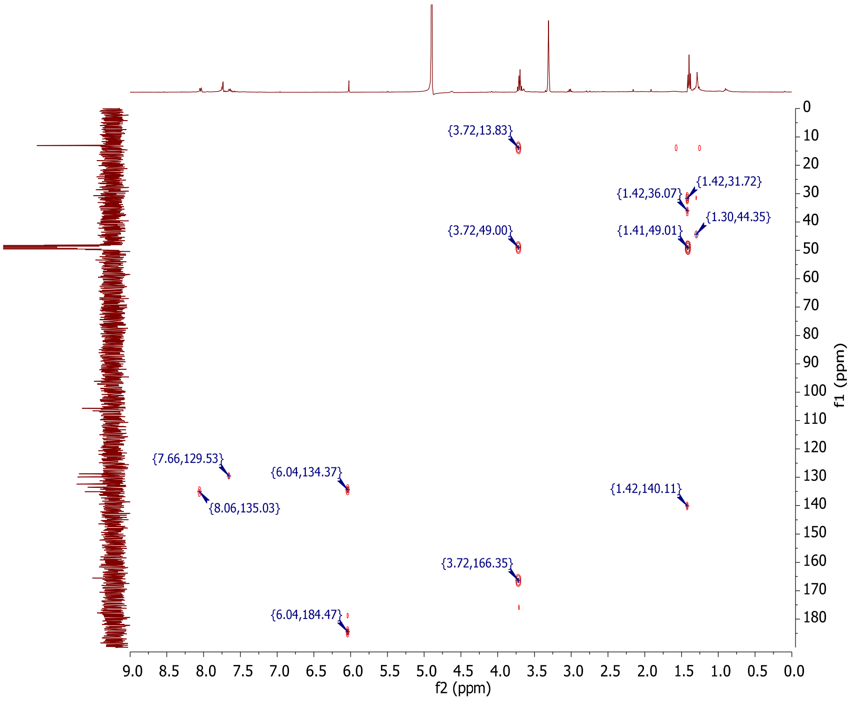

Figure S45. HMBC-NMR spectrum of compound ***4e*** in MeOD-d_4

Compound 4h:

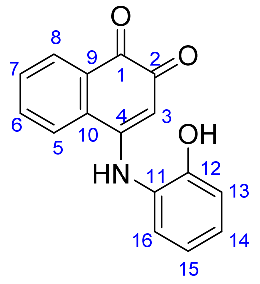

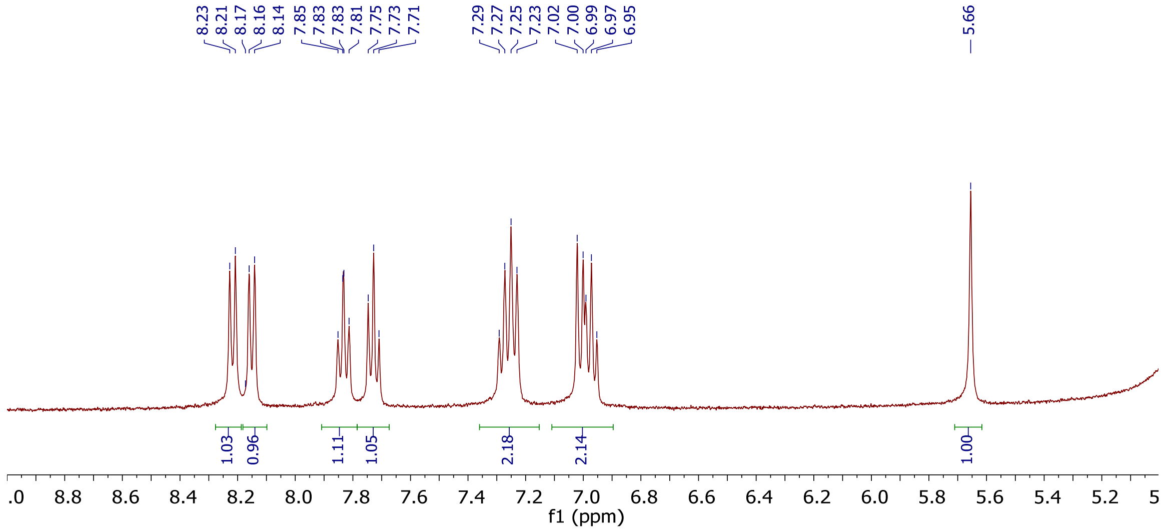

Figure S46. ^{1}H-NMR spectrum of compound ***4h*** in MeOD-d_4

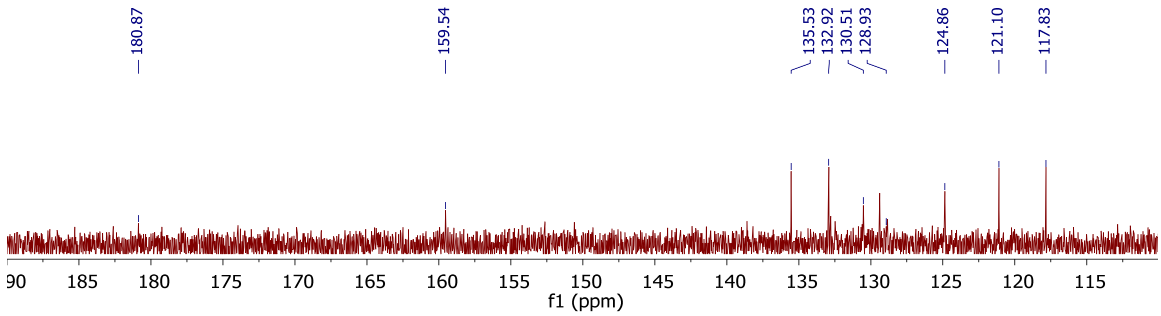

Figure S47. ^{13}C-NMR spectrum of compound ***4h*** in MeOD-d_4

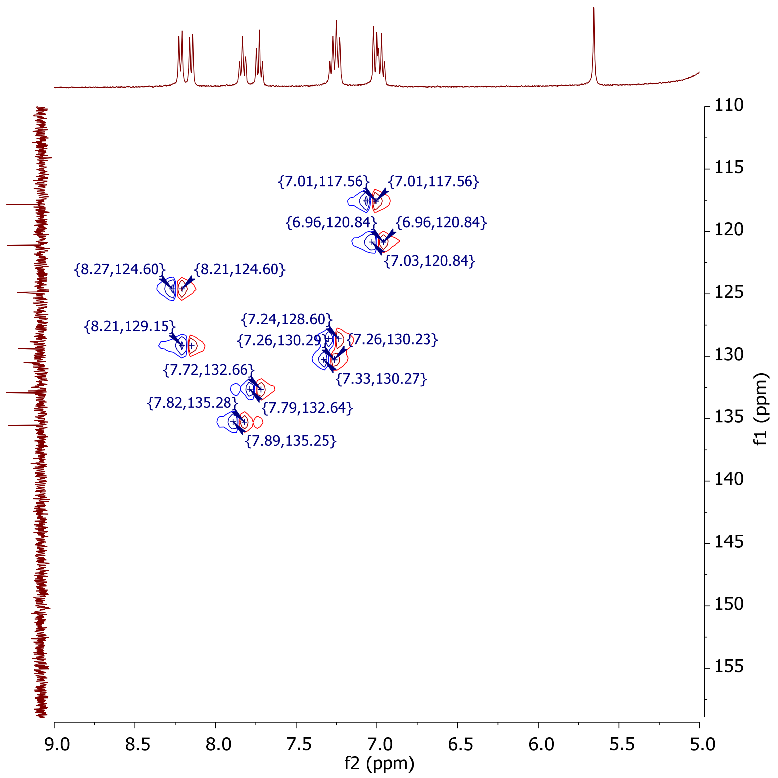

Figure S48. HSQC-NMR spectrum of compound ***4h*** in MeOD-d_4

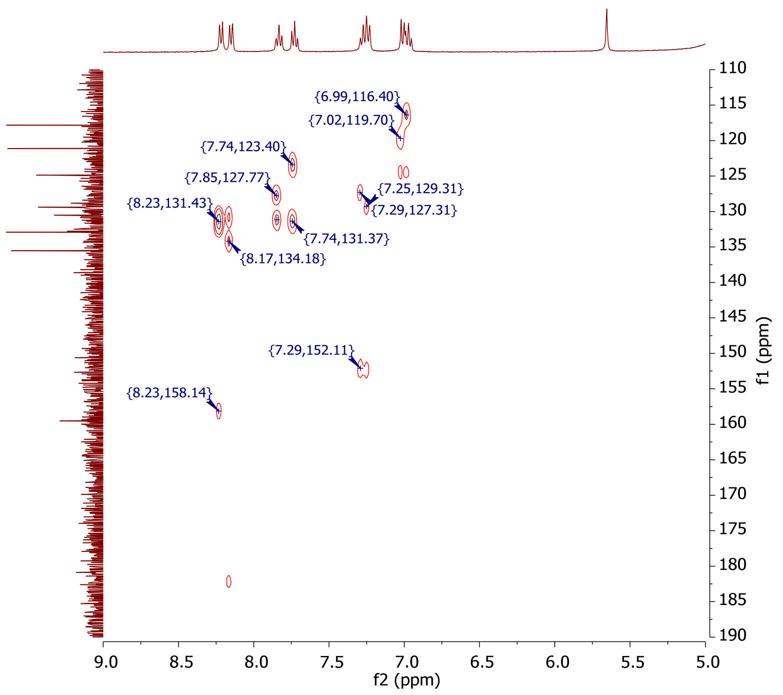

Figure S49. HMBC-NMR spectrum of compound ***4h*** in MeOD-d_4

8. ESI/MS spectra

ESI/MS spectra of compound **3*a*** ($C_{16}H_{12}N_2O_2$) MW= 264.3 g/mol

Negative mode *m/z* = 263 $[M\text{-}H]^-$; Positive mode *m/z* = 265 $[M+H]^+$;

A)

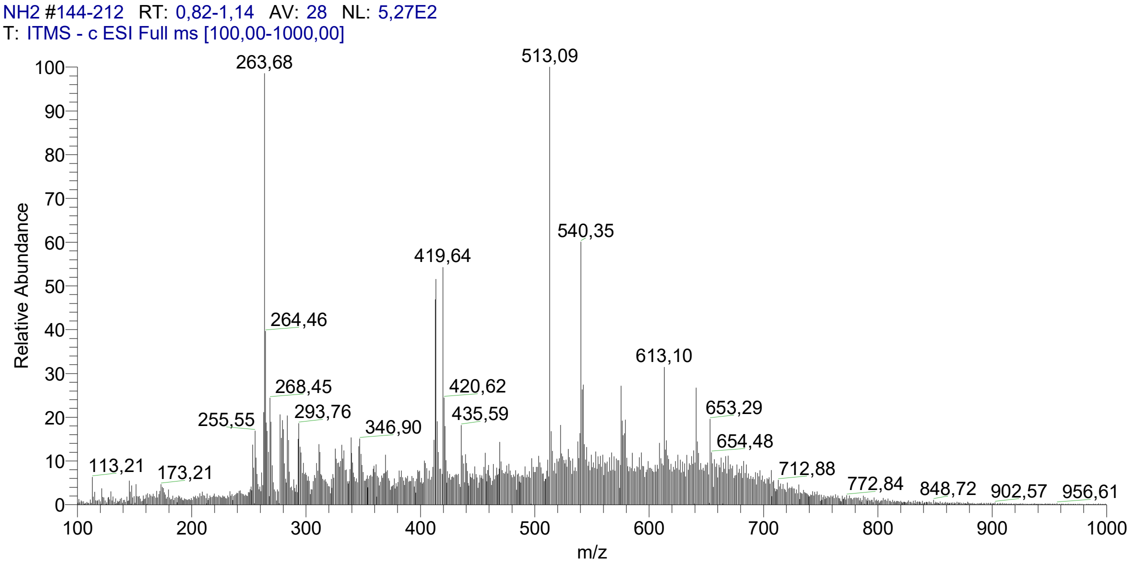

B)

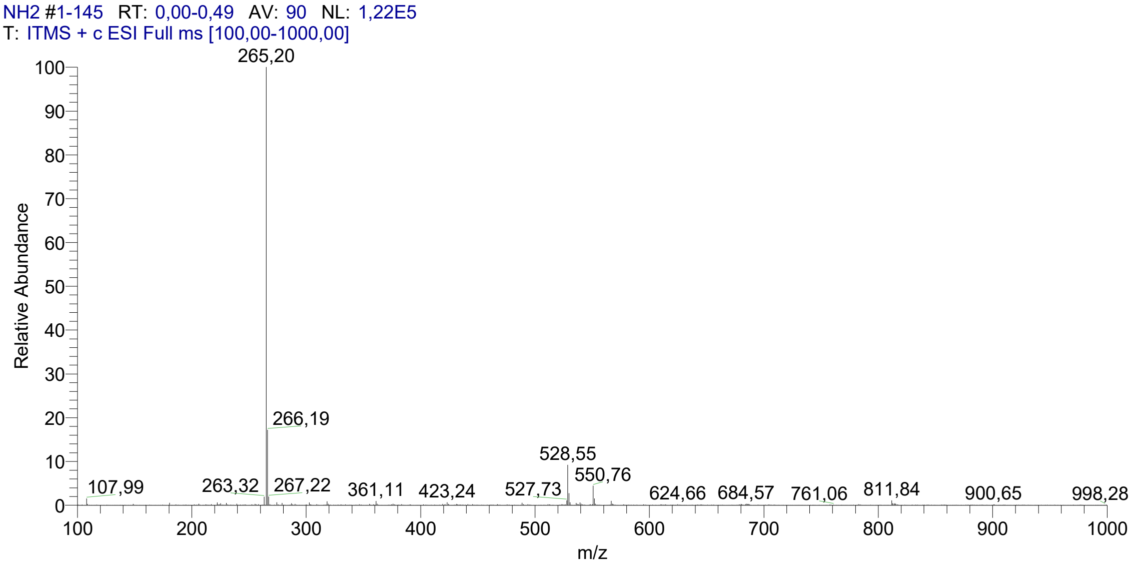

Figure S50. A) ESI(-)/MS spectrum of compound ***3a;*** C) ESI(+)/MS spectrum of compound ***3a.***

ESI/MS spectra of compound ***3b*** ($C_{16}H_{11}NO_3$) MW= 265.3 g/mol

Negative mode *m/z* = 264 $[M-H]^-$; Positive mode *m/z* = 266 $[M+H]^+$;

A)

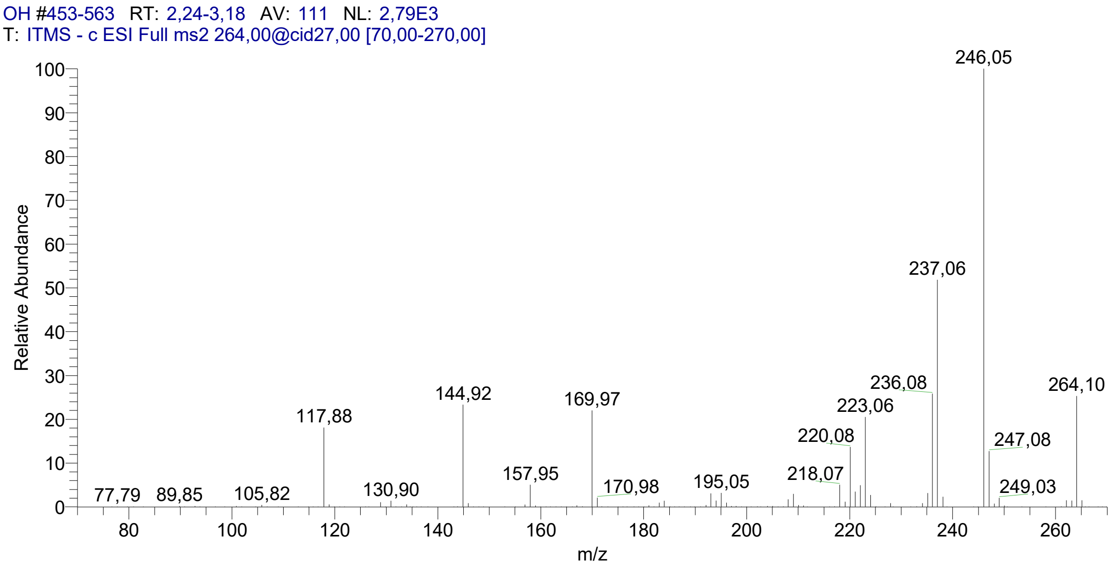

B)

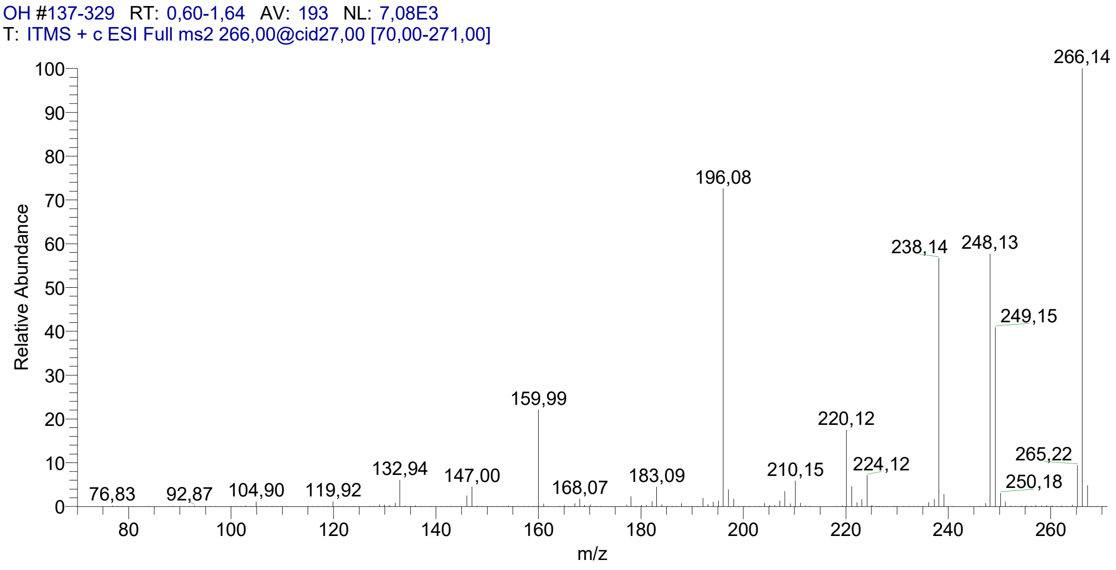

Figure S51. A) ESI(-)/MS spectrum of compound ***3b***, B)) ESI(+)/MS spectrum of compound ***3b***.

ESI/MS spectra of compound ***3c*** ($C_{16}H_{11}NO_3$) MW= 279.1 g/mol

Positive mode *m/z* = 280 $[M+H]^+$;

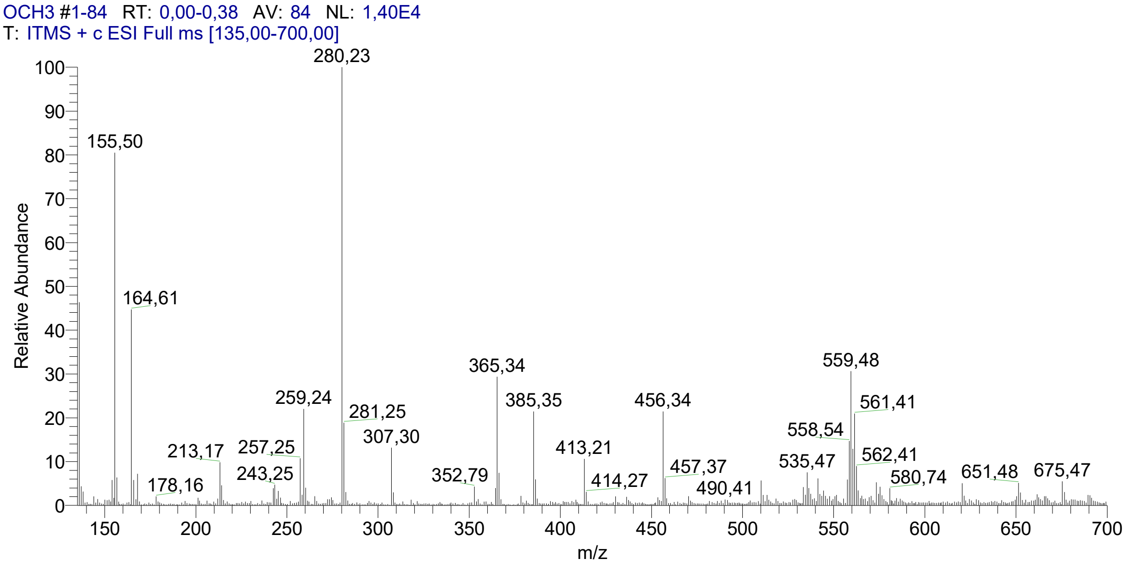

Figure S52. ESI(+)/MS spectrum of compound ***3c*.**

ESI/MS spectra of compound ***3d*** ($C_{16}H_{11}NO_3$) MW= 274.1 g/mol

Negative mode *m/z* = 273 [M-H]$^-$; Positive mode *m/z* = 275 [M+H]$^+$;

A)

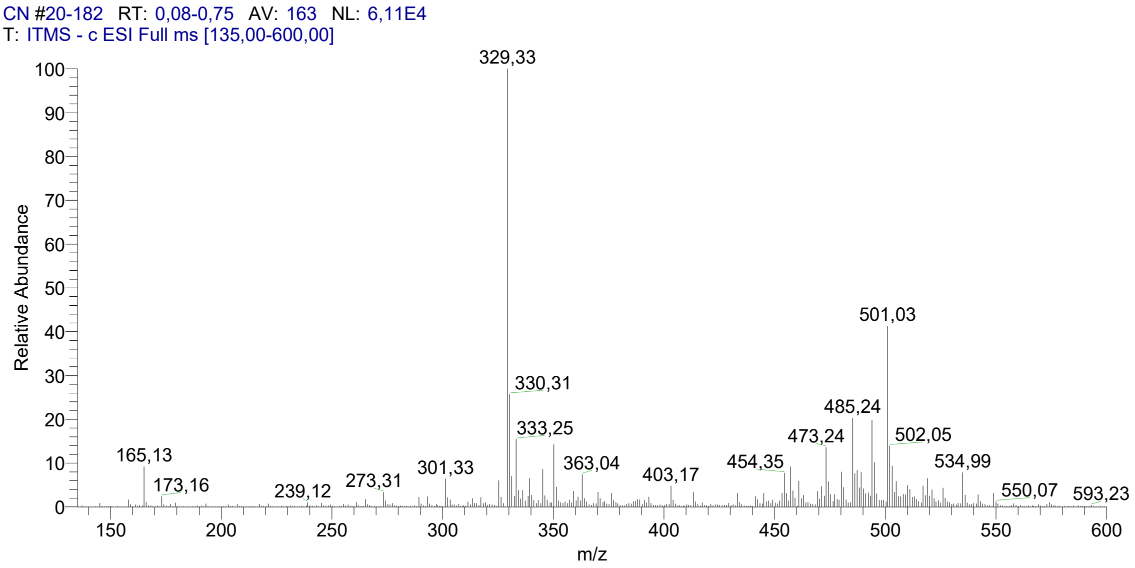

B)

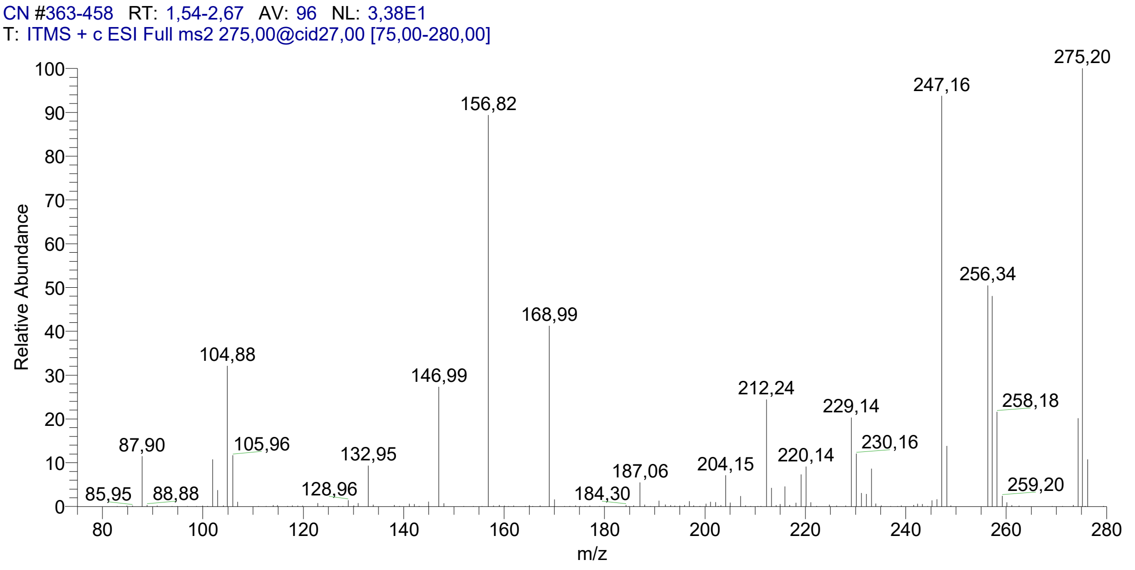

Figure S53. A) ESI(-)/MS spectrum of compound ***3d**,* B)) ESI(+)/MS spectrum of compound ***3d*.**

ESI/MS spectra of compound ***3e*** ($C_{14}H_{15}NO_2$) MW= 229.1 g/mol

Negative mode *m/z* = 228 $[M-H]^-$; Positive mode *m/z* = 230 $[M+H]^+$;

A)

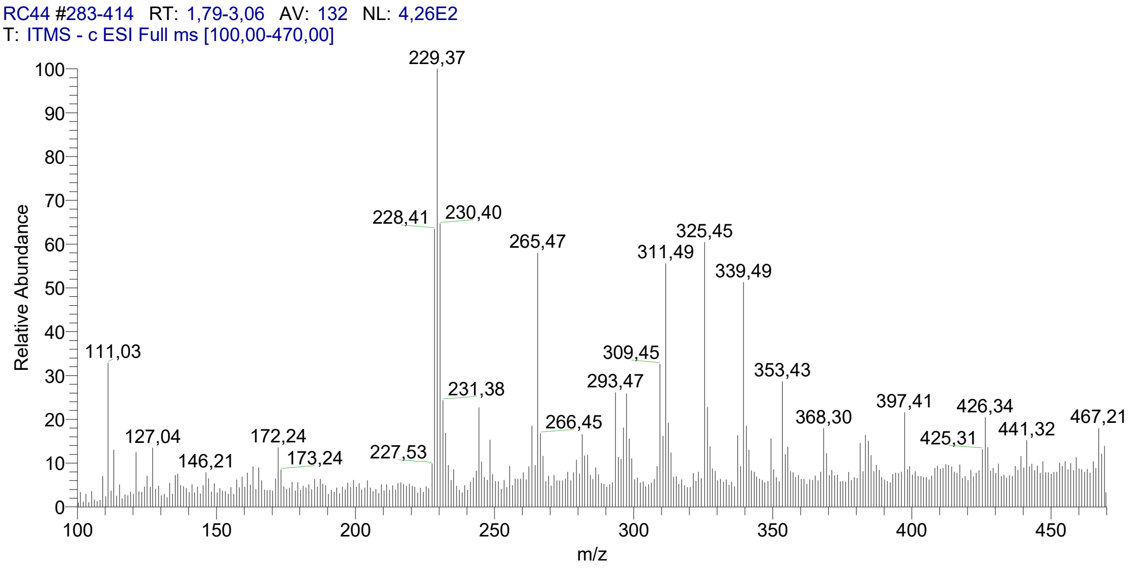

B)

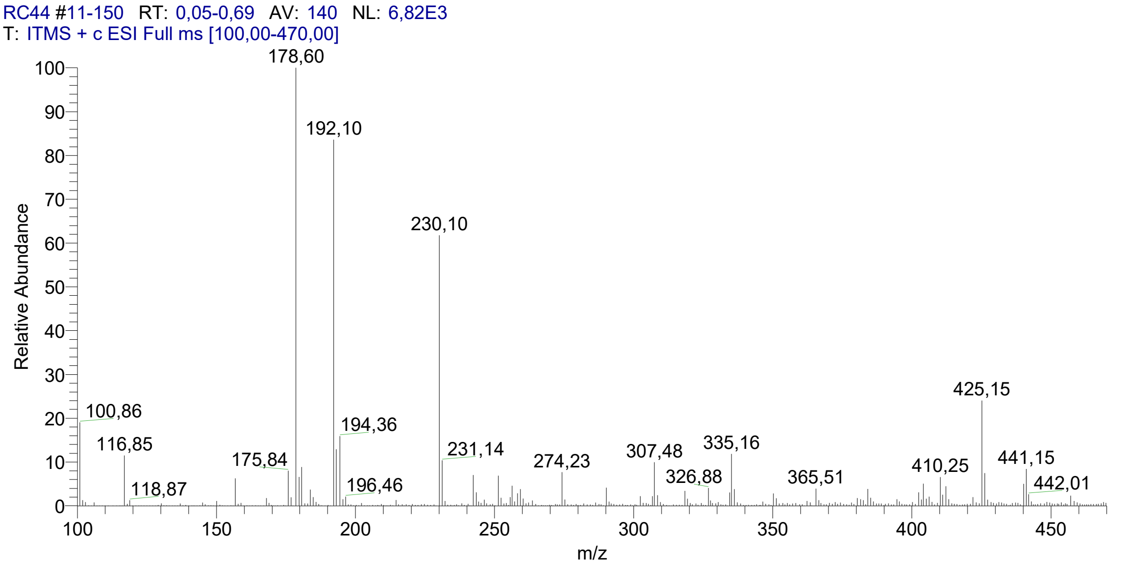

Figure S54. A) ESI(-)/MS spectrum of compound ***3e,*** B) ESI(+)/MS spectrum of compound ***3e***.

ESI/MS spectra of compound ***3f*** ($C_{14}H_{15}NO_2$) MW= 229.11 g/mol

Positive mode *m/z* = 230 $[M+H]^+$;

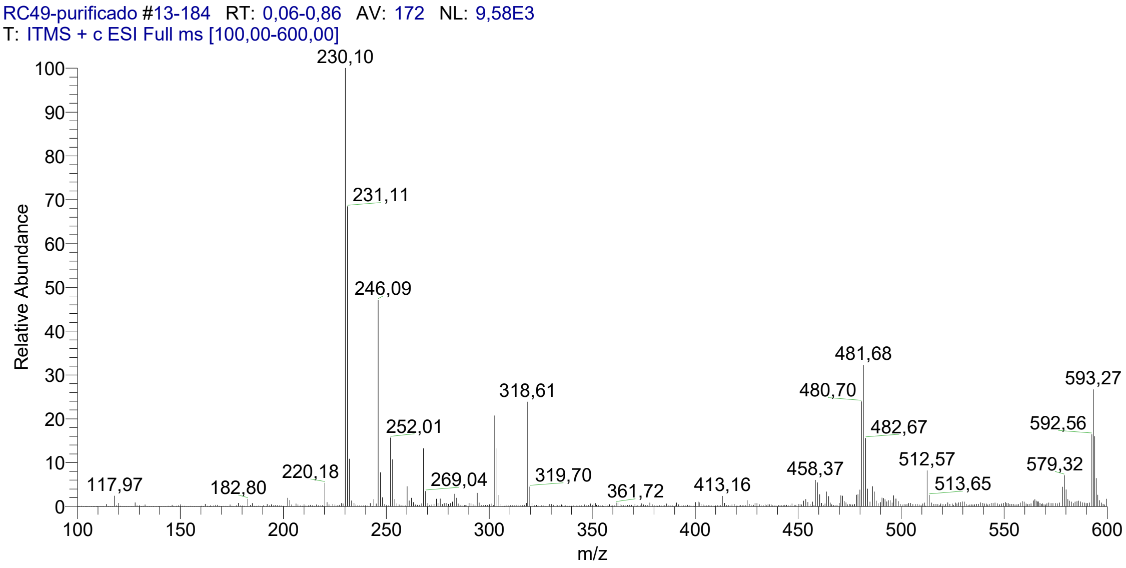

Figure S55. ESI(+)/MS spectrum of compound ***3f***.

ESI/MS spectra of compound ***4e*** ($C_{14}H_{15}NO_2$) MW= 229.1 g/mol

Negative mode *m/z* = 228 $[M-H]^-$; Positive mode *m/z* = 230 $[M+H]^+$;

A)

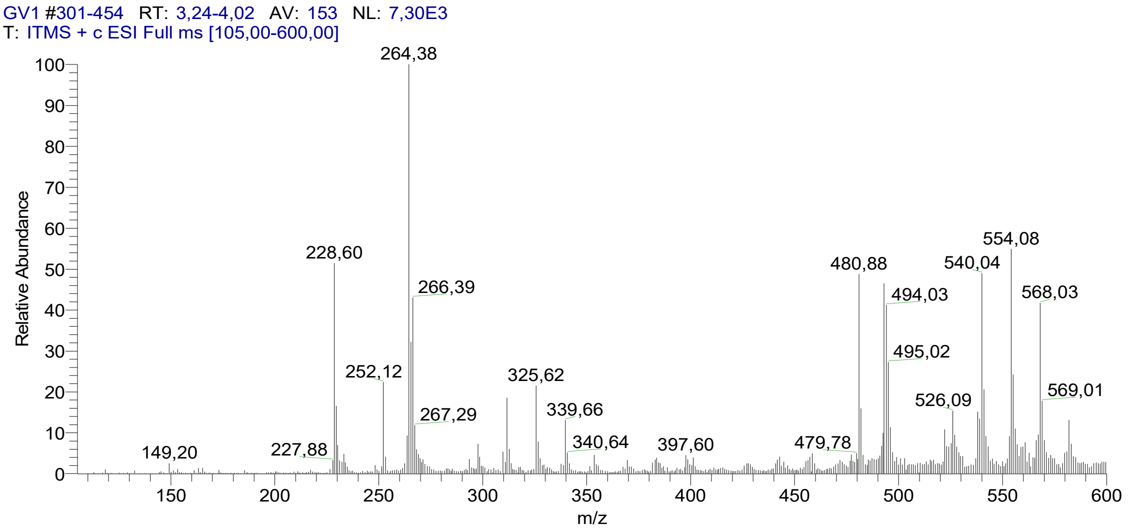

B)

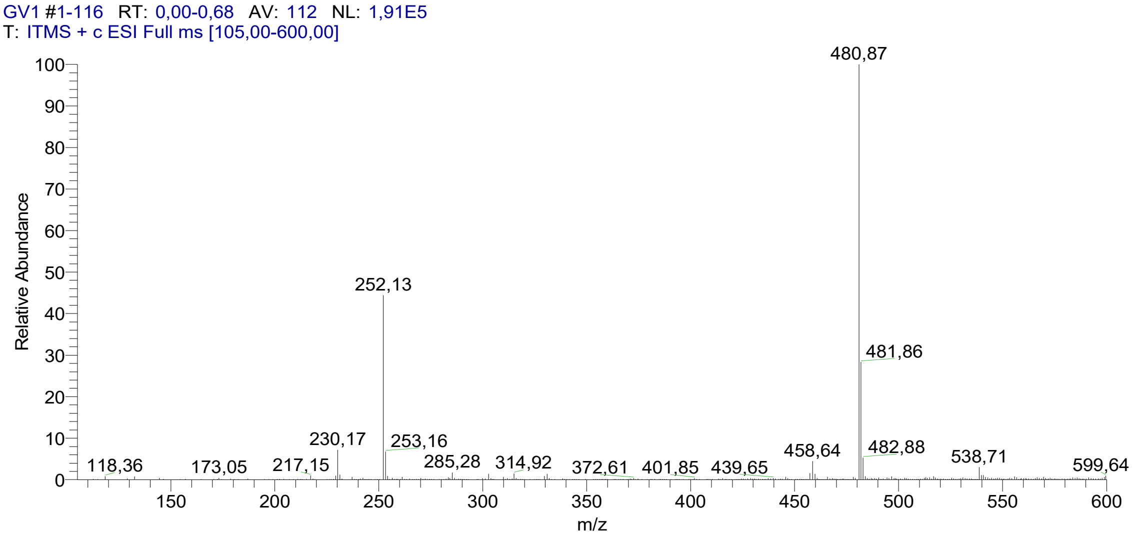

Figure S56. A) ESI(-)/MS spectrum of compound ***4e,*** B) ESI(+)/MS spectrum of compound ***4e***.

ESI/MS spectra of compound ***4f*** ($C_{14}H_{15}NO_2$) MW= 229.11 g/mol

Positive mode *m/z* = 230 $[M+H]^+$;

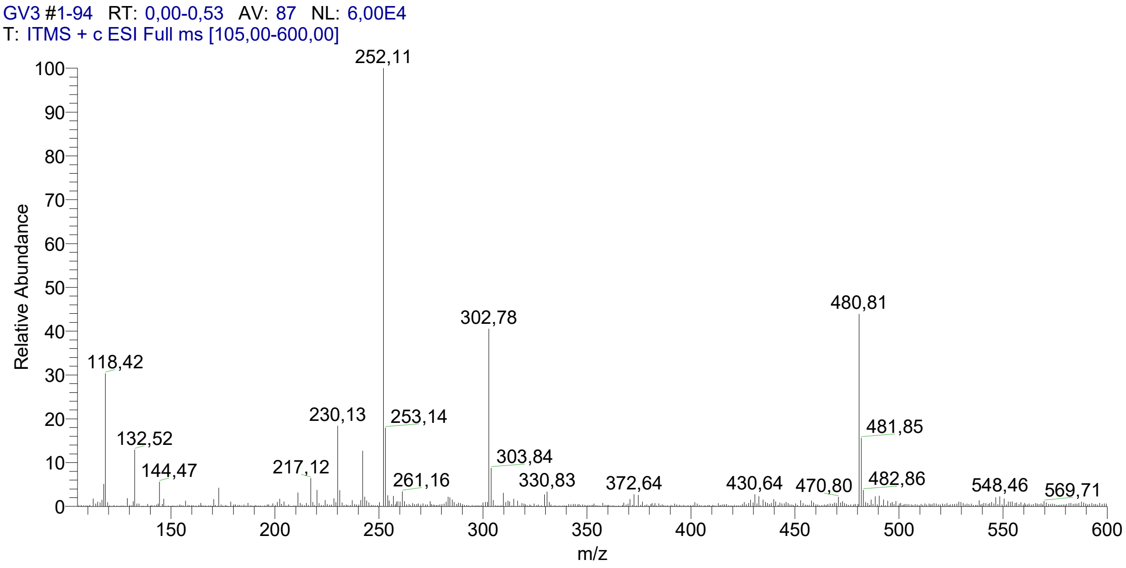

Figure S57. ESI(+)/MS spectrum of compound ***4f***.

ESI/MS spectra of compound ***4h*** ($C_{16}H_{11}NO_3$) MW= 265.3 g/mol

Negative mode *m/z* = 264 $[M-H]^-$; Positive mode *m/z* = 266 $[M+H]^+$;

A)

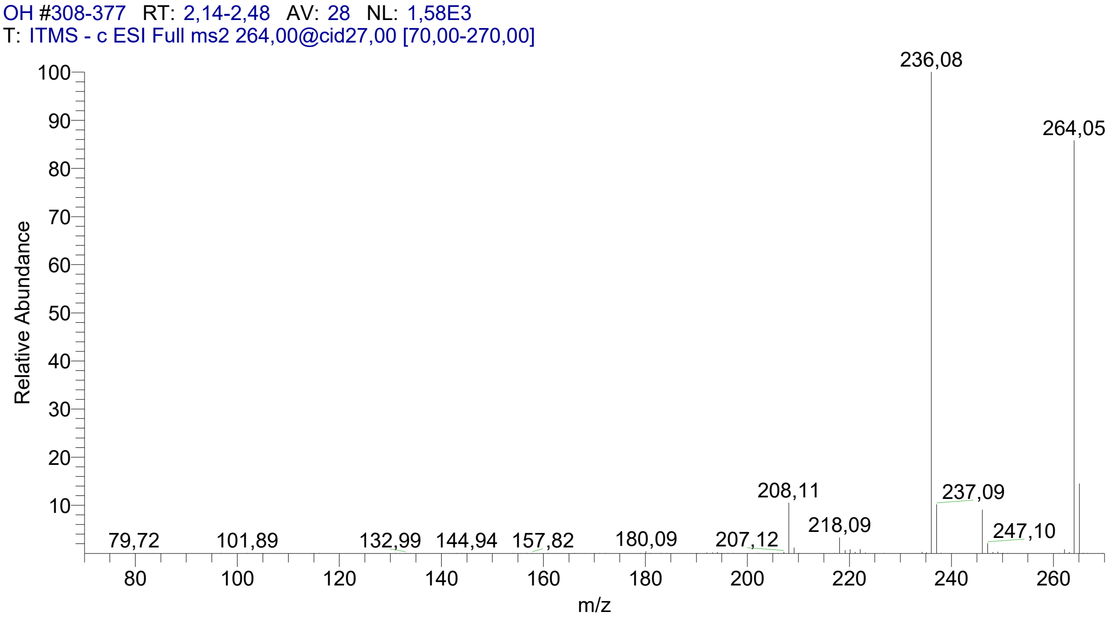

B)

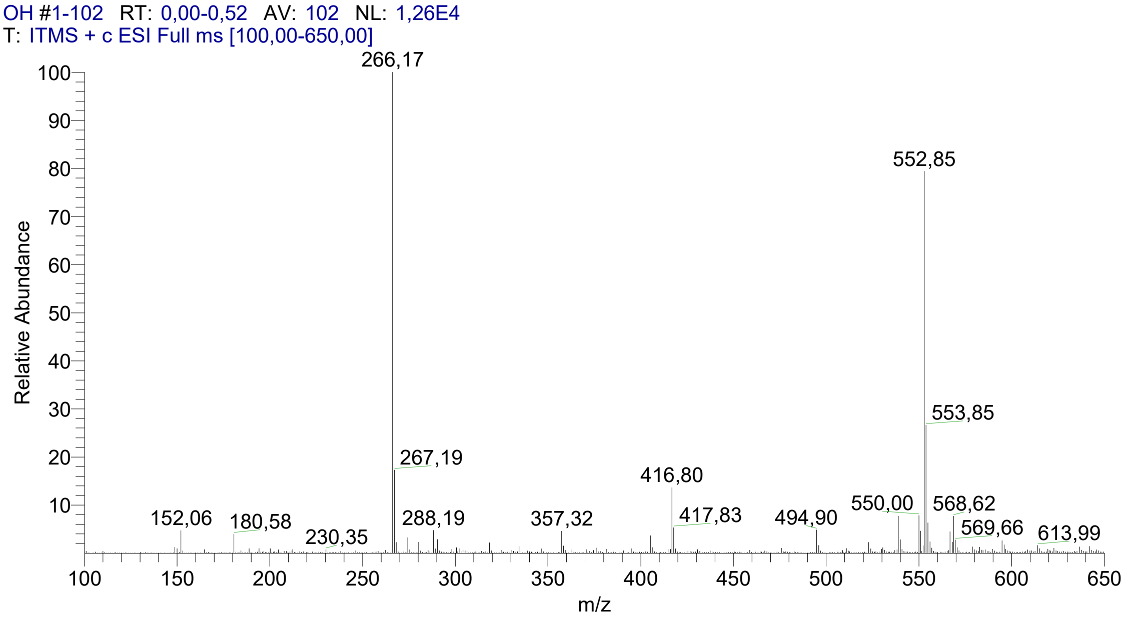

Figure S58. A) ESI(-)/MS spectrum of compound ***4h***, B)) ESI(+)/MS spectrum of compound ***4h***.

9. HRESI/TOFMS spectra

Acquisition Parameter

Source Type	ESI	Ion Polarity	Positive	Set Nebulizer	2.8 Bar
Focus	Active	Set Capillary	4500 V	Set Dry Heater	200 °C
Scan Begin	100 m/z	Set End Plate Offset	-500 V	Set Dry Gas	8.0 l/min
Scan End	1000 m/z	Set Charging Voltage	2000 V	Set Divert Valve	Waste
		Set Corona	0 nA	Set APCI Heater	0 °C

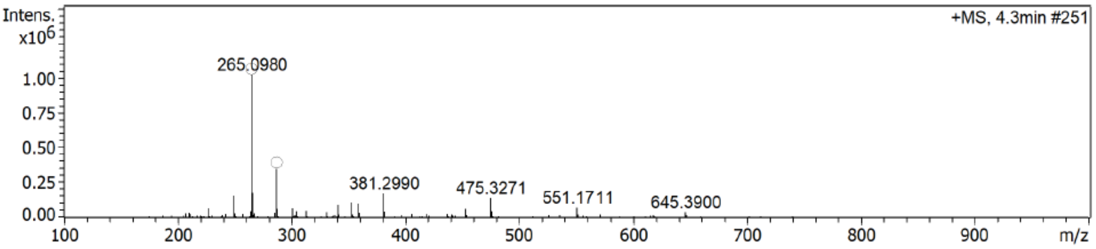

Meas. m/z	#	Ion Formula	m/z	err [ppm]	mSigma	# mSigma	Score	rdb	e⁻ Conf	N-Rule
265.0980	1	C16H13N2O2	265.0972	-3.2	4.7	1	100.00	12.0	even	ok
287.0799	1	C16H12N2NaO2	287.0791	-2.8	6.5	1	100.00	12.0	even	ok

Figure S59. HR ESI(+)/MS spectrum of compound ***3a***.

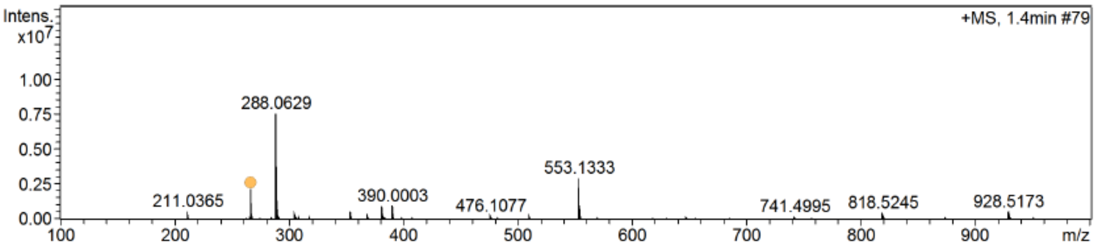

Meas. m/z	#	Ion Formula	m/z	err [ppm]	mSigma	# Sigma	Score	rdb	e⁻ Conf	N-Rule
266.0810	1	C16H12NO3	266.0812	0.7	0.7	1	100.00	11.5	even	ok

Figure S60. HR ESI(+)/MS spectrum of compound ***3b***.

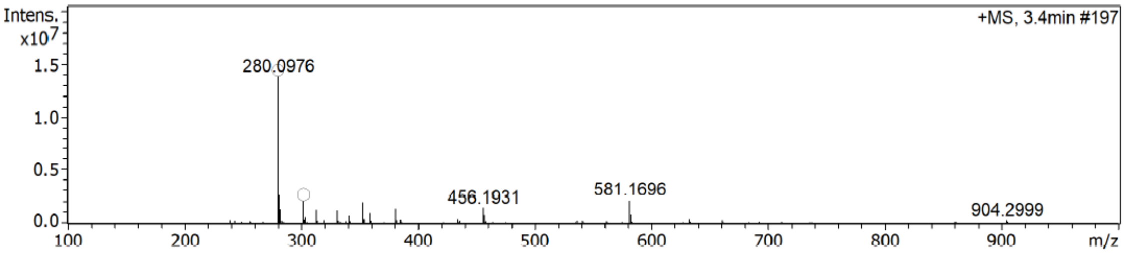

Meas. m/z	#	Ion Formula	m/z	err [ppm]	mSigma	# mSigma	Score	rdb	e⁻ Conf	N-Rule
280.0976	1	C17H14NO3	280.0968	-2.9	1.4	1	100.00	12.0	even	ok
302.0796	1	C17H13NNaO3	302.0788	-2.7	2.2	1	100.00	12.0	even	ok

Figure S61. HR ESI(+)/MS spectrum of compound ***3c*.**

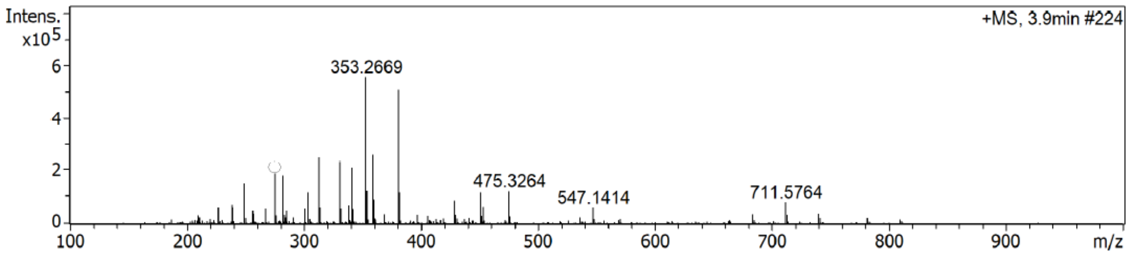

Meas. m/z	#	Ion Formula	m/z	err [ppm]	mSigma	# mSigma	Score	rdb	e⁻ Conf	N-Rule
275.0819	1	C17H11N2O2	275.0815	-1.5	7.5	1	100.00	14.0	even	ok

Figure S62. HR ESI(+)/MS spectrum of compound ***3d*.**

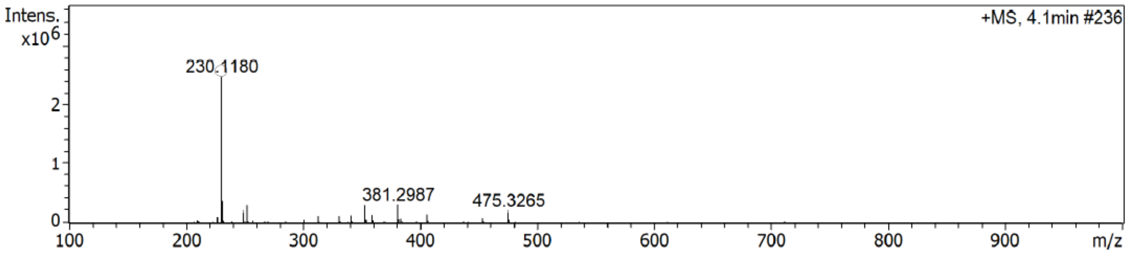

Meas. m/z	#	Ion Formula	m/z	err [ppm]	mSigma	# mSigma	Score	rdb	e⁻ Conf	N-Rule
230.1180	1	C14H16NO2	230.1176	-2.1	6.4	1	100.00	8.0	even	ok

Figure S63. HR ESI(+)/MS spectrum of compound ***3e***

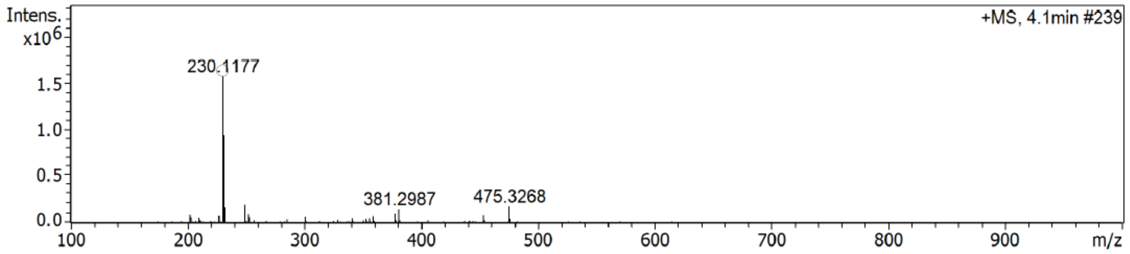

Meas. m/z	#	Ion Formula	m/z	err [ppm]	mSigma	# mSigma	Score	rdb	e⁻ Conf	N-Rule
230.1177	1	C14H16NO2	230.1176	-0.6	260.4	1	100.00	8.0	even	ok

Figure S64. HR ESI(+)/MS spectrum of compound ***3f*.**

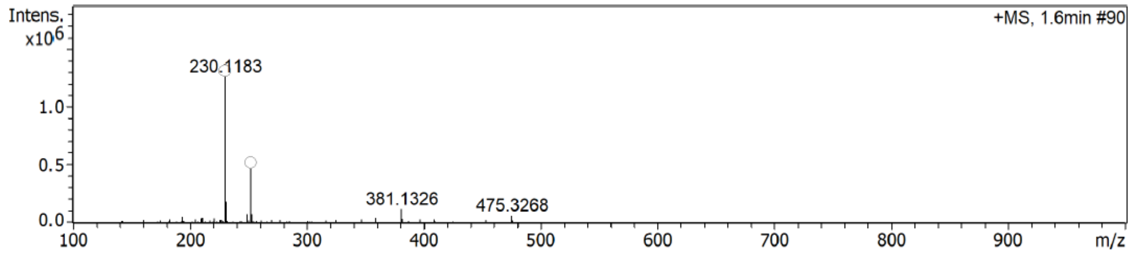

Meas. m/z	#	Ion Formula	m/z	err [ppm]	mSigma	# mSigma	Score	rdb	e⁻ Conf	N-Rule
230.1183	1	C14H16NO2	230.1176	-3.1	6.8	1	100.00	8.0	even	ok
252.1006	1	C14H15NNaO2	252.0995	-4.3	5.1	1	100.00	8.0	even	ok

Figure S65. HR ESI(+)/MS spectrum of compound ***4e***

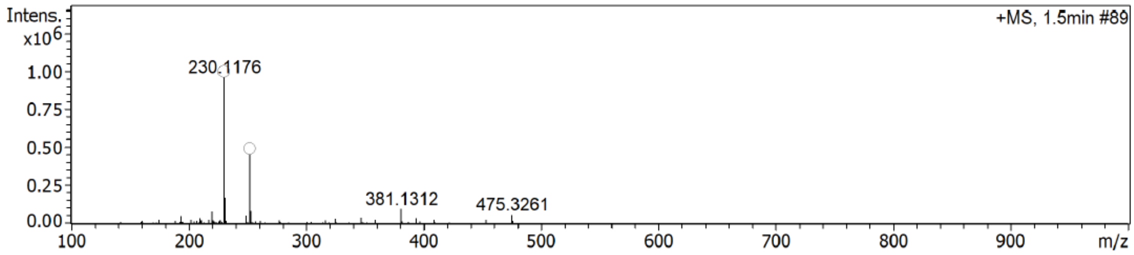

Meas. m/z	#	Ion Formula	m/z	err [ppm]	mSigma	# mSigma	Score	rdb	e⁻ Conf	N-Rule
230.1176	1	C14H16NO2	230.1176	0.0	15.6	1	100.00	8.0	even	ok
252.0997	1	C14H15NNaO2	252.0995	-0.7	14.3	1	100.00	8.0	even	ok

Figure S66. HR ESI(+)/MS spectrum of compound ***4f*****.**
